# Role of Ionic Media in f–f Transitions, Stability, and the Structure of Ln(III) Coordination Compounds

**DOI:** 10.1021/acs.inorgchem.6c02135

**Published:** 2026-08-10

**Authors:** Marcin Ziobro, Rafał Janicki

**Affiliations:** Faculty of Chemistry, 49572 University of Wrocław, F. Joliot Curie 14, 50-383 Wrocław, Poland

## Abstract

In this study, it was demonstrated that the composition of the ionic medium in solution may significantly influence not only the complex stability but also the spectroscopic properties of 4f electrons. The following reactions were investigated: [Er­(EDTA)­(H_2_O)_2_]^−^ + OH^–^ ⇄ [Er­(EDTA)­(OH)­(H_2_O)]^2–^ + H_2_O and [Er­(EDTA)­(OH)­(H_2_O)]^2–^ + M^+^ ⇄ M­[Er­(EDTA)­(OH)­(H_2_O)]^−^ (where M = Na, K, [C­(NH_2_)_3_] and [N­(CH_3_)_4_]). To identify this effect, various physical methods were employed, including crystal X-ray diffraction, EDS, high-resolution UV–vis-NIR absorption spectroscopy, ^89^Y NMR spectroscopy, potentiometry, osmometry, and ICP OES, as well as theoretical approaches such as Specific Ion Interaction Theory and York regression. Finally, it was shown that kosmotropic inorganic cations (Na^+^, K^+^) stabilize hydroxy complexes more effectively than bulky chaotropic organic cations ([C­(NH_2_)_3_]^+^ and [N­(CH_3_)_4_]^+^). As a consequence, logβ increased from 1.73 ± 0.23 (at zero ionic strength) to 3.5 ± 0.2 at I = 4 mol·kg^–1^ in the presence of KCl. The potassium cations, for which the enthalpy of hydration is lower than that of Na^+^, more readily release water molecules from their nearest coordination sphere and, as a result, may interact more directly with the [Er­(EDTA)­(OH)­(H_2_O)]^2–^ complex anion than the other cations under study, forming stable ion pairs of K­[Er­(EDTA)­(OH)­(H_2_O)]^−^. These in turn exhibit spectroscopic features different from those of [Er­(EDTA)­(OH)­(H_2_O)]^2–^.

## Graphical abstract

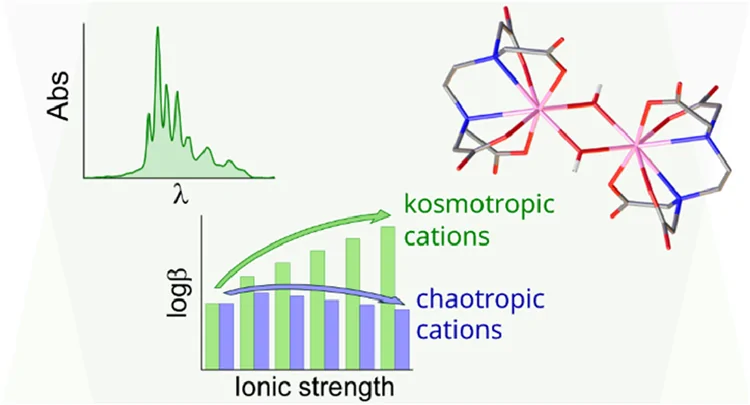

## Introduction

The f–f transitions observed in the spectra of f-block elements are often primarily interpreted in terms of changes within the nearest coordination sphere, although outer-sphere interactions may also influence the optical properties of these systems. This includes both the energies of the 4f^n^ manifold (i.e., the crystal-field splitting of ^2S+1^L_J_ levels) and the intensity of the 4f–4f transitions. The influence of the further coordination spheres on the spectroscopic features of f–f transitions is more subtle and may be observed mainly in the solid state, for example, in crystals of typical inorganic compounds, such as oxides and halides, which are characterized by a low phonon energy lattice, in which Ln­(III) ions are in close proximity to each other.^1,2^ The phase transition caused by the partial rotation of outer-sphere ions/molecules may also influence the spectroscopic properties of Ln­(III) ions, as demonstrated for [C­(NH_2_)_3_]_3_[Dy­(EDTA)­(CO_3_)]·H_2_O crystals.^3^ Even more subtle interactions, such as isotopic structures in the absorption and luminescence spectra of ^6^Li_*x*_^7^Li_1–*x*_YF_4_:Ho­(III), arise from the coupling between the nuclear spins of ^6^Li and ^7^Li and the crystal-field states of Ho­(III).^4^ Nevertheless, the observation of such subtle effects requires high-resolution UV–vis-NIR spectroscopy. Otherwise, low-resolution spectra may lead to incorrect identification of electronic energy levels.^5^ The influence of the further coordination sphere on the spectroscopy of Ln­(III) compounds in solution is challenging to observe. Many different effects may overlap with those mentioned above for the solid state, including dynamic effects associated with changes in the molecular geometry of the complex, as well as the coexistence of species with various stoichiometries and coordination numbers.^6,7^ This seriously complicates the analysis and interpretation of experimental data.

Out of the physical methods applied for the analysis of chemical equilibria in solutions containing f-element systems, high-resolution UV–vis–NIR spectroscopy is particularly useful.^8–16^ A direct comparison of the spectra of the f–f transitions of monocrystals containing structurally well-defined coordination complexes with the corresponding spectra of solutions may give valuable insights into the stoichiometry, coordination number of the metal cation, and structure of the complex in solution.^17–19^ No additional manipulations/normalizations of the intensity of bands are required to apply this method for the qualitative and quantitative analyses of systems in different phases/conditions.

High thermodynamic stability of ethylenediaminetetraacetic acid with various metal ions makes it one of the most important chelate agents used in several processes in the nuclear industry (i.e., decontamination of facilities). Thus, the EDTA ligand may be present to some extent in liquid nuclear fuel.^20,21^ Recent studies on the PuO_2_ and Pu­(OH)_3_ solubility in EDTA solutions in the presence of Ca­(II) and/or Fe­(OH)_3_ suggest that the environmental mobility of plutonium may significantly increase due to Pu­(IV/III)–EDTA and Pu­(IV/III)–OH–EDTA complex formation.^22^ Although there is no doubt regarding the thermodynamic stability of the An­(IV/III)/Ln­(III)–EDTA systems (eq 1), the formation of the hydroxy species [M­(EDTA)­OH]^n–^ has not been sufficiently documented in the literature (see Table 1 and the references cited therein).

1 $${[M\left(\mathrm{EDTA}\right)\left(H_{2}O{)}_{n}\right]}^{n-}+OH^{-}⇄[M\left(\mathrm{EDTA}\right)(H_{2}O{)}_{n}{\left(\mathrm{OH}\right)]}^{\left(n+1\right)-}$$

The wide scattering of the formation constants (β_OH_) of reaction ([Disp-formula eq1])

2 $$\beta_{\mathrm{OH}}=\frac{[M\left(\mathrm{EDTA}\right)\left(H_{2}O{)}_{n}\left(\mathrm{OH}\right)\right]}{[M\left(\mathrm{EDTA}\right)\left(H_{2}O{)}_{n}\right]\cdot \left[\mathrm{OH}\right]}$$

for both lanthanide­(III) and actinide­(III) cations (Table 1), well epitomize this problem.

**Table 1:** Literature Data for Hydrolysis Reaction (1)

[M(EDTA)(OH)]^−^	logβ_OH_	I/M	method	ref
Sc(III)	3.4		^1^H NMR	^23^
Y(III)	2.1		^1^H NMR	^23^
La(III)	<3		^1^H NMR	^23^
Pr(III)	1.14		^1^H NMR	^24^
Nd(III)	1.78		UV–vis	^25^
Eu(III)	1.52		^1^H NMR	^24^
Eu(III)	1.36		polarography	^26^
Eu(III)	4.87	0	solubility/ICP-MS	^27^
Gd(III)	3.61 ± 0.06	0.5	potentiometry	^28^
Er(III)	3.40 ± 0.13	3.5	UV–vis	^29^
Er(III)	3.56 ± 0.21	3.5	potentiometry	^29^
Yb(III)	1.7		^1^H NMR	^24^
Lu(III)	2.3		^1^H NMR	^23^
Pu(III)	<3	0	solubility/ICP-MS	^22^
Am(III)	∼2			^30^
Am(III)	2.62 ± 0.13	0	electromigration	^31^
Cm(III)	4.0 ± 1.9	0	TRLFS	^22^

Table 1 reveals that the values of the formation constant of reaction 1 may differ significantly even for systems containing the same M­(III) cation; moreover, these results seem to depend on the implemented experimental method. Consequently, the pH range of the solutions at which the concentration of hydroxy complex [Eu­(EDTA)­(OH)]^2–^ predominates is between 9.13 and 12.48.

To determine the thermodynamic stability of the complex in solutions, it is necessary to refer to the experimental results in the standard state. According to the Nuclear Atomic Energy recommendation, the Specific Ion Interaction model presented for the first time by Brønsted^32^ and extended later by Guggenheim^33^ and Scatchard,^34^ should be used to extrapolate the experimental conditional stability constants to the zero ionic state using eq ([Disp-formula eq3]).^35^

3 $$\ln\beta_{\mathrm{ML}}+A_{\gamma }\Delta z_{\mathrm{RS}}^{2}\frac{\sqrt{I_{m}}}{1+1.5\sqrt{I_{m}}}=\ln\beta_{\mathrm{ML}}^{0}+{\Delta \varepsilon }_{\gamma (\mathrm{RS})}m_{\mathrm{ij}}$$

where ln β_*ML*_ and ln β_*ML*_^0^ are the conditional and extrapolated to zero ionic strength stability constants, respectively; $A_{\gamma }=1.172\;kg^{1/2}\cdot {\mathrm{mol}}^{-1/2}$ (at 298 K, 1 bar) is the Debye–Hückel limiting slope, a constant at a given temperature and solvent, for any electrolyte; Δ*z*_RS_^2^ is the difference in the squared charges of the reacting species; *I*_m_ is the ionic strength of the solution; Δε_γ(RS)_ is the difference in the interaction parameter of the reacting species, representing short-range specific interactions between ions and is a key quantity describing deviations from ideal behavior in electrolyte solutions; and *m*_*ij*_ is the molality of electrolyte *ij*, where *i* and *j* denote the constituent ions of the supporting electrolyte.

Derived in this way, the interaction parameters Δ*ε*_γ_ are often burdened with experimental inaccuracy; thus, the error of estimation -*σε*_*γ*_ significantly overestimates the ε_γ_ value.^35^

The other method of determination of the ε_γ_ interaction parameter is based on the osmotic coefficient measurements of solutions containing a pure solute. Osmotic coefficient measurements, particularly those based on freezing-point depression methods, generally provide more precise determinations of the ε_γ_ interaction parameters compared to the values derived from concentration-dependent equilibrium constants within the SIT model, owing to the lower propagation of systematic uncertainties.^33,36^ To the best of our knowledge, there are no ε_γ_ parameters for molecular Ln­(III) systems derived from the osmotic coefficient measurements that can be used to extrapolate more precisely the conditional stability constants to zero ionic strength. The correct values of the ε_γ_ interaction parameter are of key importance in the estimation of the ionic medium effect on the stability of metal complexes in solution. Moreover, the determined values of ε_γ_ for lanthanide­(III) systems may often represent the only reliable basis for estimating the initial ε_γ_ parameters for actinide­(III) compounds, as handling sufficiently concentrated solutions of actinide­(III) systems is difficult due to their high radioactivity.

Taking into account all these issues, we studied the structural, UV–vis–NIR spectroscopic, and thermodynamic aspects of the hydrolysis of the Er­(III)–EDTA systems at different ionic strengths and at a high pH of the solution. The selection of Er­(III) was motivated by its UV–vis absorption spectrum, which contains numerous f–f transitions that are particularly sensitive to changes in the Er­(III) coordination environment. To investigate the structure and speciation of the Er­(III)–EDTA system the following physical methods were applied: (i) X-ray diffraction of monocrystals to determine the molecular structure of the complex in the solid state, (ii) high-resolution UV–vis–NIR absorption spectroscopy to compare the spectral features of the f–f transitions of the Er­(III)–EDTA complex in solution and in the corresponding monocrystals to determine the molar fractions of aqueous species, (iii) ^89^Y NMR spectroscopy to distinguish the coordination number of the Ln­(III) ion in the [Ln­(EDTA)­(OH)_n_]^−(1+n)^ complex; (iv) pehametry together with UV–vis–NIR spectroscopy to determine the stability constants β_OH;_ (v) osmometry to determine the accurate values of the interaction parameters *ε*_γ_ for K^+^, Na^+^, [C­(NH_2_)_3_]^+^, and [N­(CH_3_)_4_]^+^ −Ln­(EDTA)^−^ systems.

Because hydroxo ligands readily induce the polymerization of f-element complexes, the isolation of monomeric hydroxo species is often difficult. For this reason, we decided to use the isoelectronic fluoride anion, which exhibits donor–acceptor properties similar to those of OH^–^ but, at the same time, shows a lower tendency to form polymeric species. Thus, monomeric fluoride complexes can be isolated and used as structural and spectroscopic models that may mimic hydrolyzed species.

## Experimental Section

Materials and sample preparation. Na­[Er­(EDTA)­(H_2_O)_3_]·3.5H_2_O and K­[Er­(EDTA)­(H_2_O)_2_]·4H_2_O crystals were synthesized by dissolving Er_2_O_3_ (99%, Aldrich Chem. Co., 0.05 mol) in HCl (p.a., Eurochem BGD). After complete dissolution, H_4_EDTA (99%, Chempur; 0.10 mol) was added. The mixture was heated at approximately 95 °C for 1 h, after which a solution of the appropriate alkali metal hydroxide (NaOH Chempur, KOH Eurochem BGD) was added until the final pH reached 8. Pink crystals formed after a few weeks. In a similar way, the crystals of Na­[Y­(EDTA)­(H_2_O)_3_]·5H_2_O were synthesized. K_5_[Er_2_(EDTA)_2_(OH)_2_]­Cl·5.5H_2_O crystals were synthesized by dissolving 0.005 mol of ErCl_3_·6H_2_O in water, followed by the addition of 0.005 mol of H_4_EDTA. The solution was heated, and KOH pellets were added until the final pH reached approximately 13. The reaction was performed under an inert (N_2_) atmosphere. The solution was then placed in a desiccator. After six months, light pink crystals crystallized.

[C­(NH_2_)_3_]_2_[Er­(EDTA)­(H_2_O)­(F)]·2H_2_O crystals were synthesized by dissolving 0.01 mol of K­[Er­(EDTA)­(H_2_O)_2_]·4H_2_O crystals in water. Next, an aqueous solution containing 0.005 mol of [C­(NH_2_)_3_]_2_CO_3_ (>99%, Fluka) was mixed with a 48% HF solution (Sigma-Aldrich) in a 1:2 molar ratio, and the resulting solution was mixed with a solution of K­[Er­(EDTA)­(H_2_O)_2_]. Pink, well-shaped crystals were grown after 3 weeks.

[C­(NH_2_)_3_]_3_[Er­(EDTA)­(F)_2_]·H_2_O crystals were synthesized by dissolving Er_2_O_3_ (99%, Aldrich Chem. Co., 0.002 mol) in HCl (Eurochem BGD). After complete dissolution, H_4_EDTA (99%, Chempur; 0.004 mol) was added. The mixture was heated at approximately 95 °C for 1 h, after which solid [C­(NH_2_)_3_]_2_CO_3_ (>99%, Fluka) was added until the final pH reached ∼7. Subsequently, an aqueous solution containing 0.005 mol of [C­(NH_2_)_3_]_2_CO_3_ (>99%, Fluka) was mixed with a 48% HF solution (Sigma-Aldrich) in a 1:2 molar ratio, and the resulting solutions were mixed.

K_4_[Er_2_(EDTA)_2_(F)_2_]·8H_2_O crystals were synthesized by dissolving 0.005 mol of K­[Er­(EDTA)­(H_2_O)_2_]·4H_2_O crystals in water. Next, solid KF (POL-AURA 99%, 0.005 mol) was added, and the solution was heated at ∼95 °C for 0.5 h. Pink, well-shaped crystals were obtained after 3 weeks.

The Y­(ClO_3_) solution was synthesized by dissolving Y_2_O_3_ (>99%, Merck) in HClO_4_ (Chempur 70%). The mixture was heated to 95 °C to ensure the complete dissolution of the solid.

[C­(NH_2_)_3_]_2_SO_4_ crystals were obtained by the reaction of [C­(NH_2_)_3_]_2_CO_3_ (>99%, Fluka; 0.23 mol) with diluted H_2_SO_4_ (96%, 0.23 mol) in a 1:1 molar ratio. The mixture was stirred for a few minutes. Well-formed colorless crystals were obtained after 5 weeks.

The [C­(NH_2_)_3_]­OH aqueous solution was prepared by the gradual addition of [C­(NH_2_)_3_]_2_SO_4_ (0.1 mol) dissolved in water to a boiling mixture of Ba­(OH)_2_·H_2_O (Fluka; 0.1 mol). After the reaction was complete, the mixture was centrifuged. The supernatant solution of [C­(NH_2_)_3_]­OH was collected.

The concentrations of the NaOH (Eurochem), KOH (Eurochem), [N­(CH_3_)_4_]­OH·5H_2_O (Alfa Aesar 98%), and [C­(NH_2_)_3_]­OH solutions were determined by titration with potassium hydrogen phthalate using thymolphthalein as an indicator.

A 77.2 mM mother solution of [Er­(EDTA)­(H_2_O)_2_]^−^ was prepared by dissolving the relevant amount of Na­[Er­(EDTA)­(H_2_O)_3_]·3.5H_2_O/K­[Er­(EDTA)­(H_2_O)_2_]·4H_2_O crystals in water. Solutions of [Er­(EDTA)­(H_2_O)_2_]^−^ used for spectroscopic measurements were prepared by dilution of the appropriate volume of mother solution (0.077 M [Er­(EDTA)­(H_2_O)_2_]^−^) with a relevant amount of NaCl (p.a., Eurochem BGD), KCl (p.a., Eurochem BGD), [N­(CH_3_)_4_]­Cl (>98%, Fluka AG), and [C­(NH_2_)]Cl (>98%, Fluka AG) for ionic strength adjustment (0.1 M, 0.5 M, 1 M, 2 M, 3 M, 4 M).

All water solutions were prepared with freshly deionized water (0.05 μS/cm). The experimental and calculated mole fractions of the [Er­(EDTA­(H_2_O)_2_]^−^ species as a function of pH in different electrolyte media are presented in Figures S1–S5.

For ^89^Y NMR spectroscopic measurements, Na­[Y­(EDTA)­(H_2_O)_3_] solutions (15.5 mM) were prepared in D_2_O at pH 7 and 12.5, with the ionic strength adjusted to I = 2.5 M using NaCl and KCl. A 3 M Y­(ClO_4_)_3_ solution in D_2_O was used as the standard (Figure S6).

## Crystal Structure Determination

Suitable crystals were selected or cut from larger ones, mounted on a diffractometer equipped with a CCD counter, and measured at 100 K. The structures were solved routinely by using indirect phasing. The C- and N-bonded hydrogen atoms were placed in positions calculated from geometry, and those bonded to O atoms were found from the difference Fourier maps or were placed based on chemical reasoning. The final refinement was anisotropic for all of the non-H atoms. The computations we performed with the SHELXT^37^ and SHELXL^38^ programs, and molecular graphics were prepared with Olex2.^39^

C_10_H_24_ErKN_2_O_14_ (CCDC 2542954; ErW2), *M* = 602.67, monoclinic, space group *P*2/*n*, *Z* = 4, *a* = 8.659(2), *b* = 25.821(3), *c* = 8.722(2) Å, β = 97.54(2)°, *V* = 1933.2(7) Å^3^, F(000) = 1188, μ = 4.63 mm^–1^, *D*_c_ = 2.071 g·cm^–3^, crystal size = 0.326 × 0.189 × 0.085 mm, θ = 3.5–30.3°, index range −11 ≤ *h* ≤ 12, −36 ≤ *k* ≤ 36, −12 ≤ *l* ≤ 12. Final *R* indices: *R*_int_ = 0.018, *R*[*F* ^2^ > 2σ­(*F* ^2^)] = 0.021, w*R*(*F* ^2^) = 0.041, *S* = 1.19. Largest diffraction peak and hole: 0.64 and −0.91 e·Å^–3^ (Table S1).

C_10_H_25_ErN_2_NaO_14.5_ (CCDC 2542955; ErW3), *M* = 595.57, orthorhombic, space group *Fdd*2, Z = 16, *a* = 34.665(6), *b* = 18.746(4), *c* = 12.040(2) Å, *V* = 7824.1(3) Å^3^, *F*(000) = 4704, μ = 4.388 mm^–1^, *D*_c_ = 2.022 g·cm^–3^, crystal size = 0.202 × 0.130 × 0.096 mm, θ = 3–30.5°, index range −49 ≤ *h* ≤ 49, −24 ≤ *k* ≤ 26, −17 ≤ *l* ≤ 16. Final *R* indices: *R*_int_ = 0.053, *R*[*F* ^2^ > 2σ­(*F* ^2^)] = 0.027, w*R*(*F* ^2^) = 0.059, *S* = 1.03. Largest diffraction peak and hole: 1.33 and −1.38 e·Å^–3^ (Table S1).^40,41^

C_12_H_30_ErFN_8_O_11_ (CCDC 2542956; ErWF), *M* = 648.70, triclinic, space group P1̅, *Z* = 2, *a* = 9.517(6), *b* = 11.828(8), *c* = 12.237(8) Å, α = 64.08(7)°, β = 70.05(6)°, γ = 68.09(6)°, *V* = 1122.9(15) Å^3^, *F*(000) = 646, μ = 4.388 mm^–1^, *D*_c_ = 1.919 g·cm^–3^, crystal size = 0.470 × 0.338 × 0.055 mm, θ = 3.2–28.9°, index range −10 ≤ *h* ≤ 12, −15 ≤ *k* ≤ 16, −15 ≤ *l* ≤ 15. Final *R* indices: *R*_int_ = 0.021, *R*[*F* ^2^ > 2σ­(*F* ^2^)] = 0.019, w*R*(*F* ^2^) = 0.044, *S* = 1.07. Largest diffraction peak and hole: 0.89 and −0.66 e·Å^–3^ (Table S1).

C_13_H_32_ErF_2_N_11_O_9_ (CCDC 2542952; ErF2), *M* = 691.75, monoclinic, space group *P*2_1_/*n*, *Z* = 4, *a* = 13.411(3), *b* = 10.408(2), *c* = 17.509(3) Å, β = 101.19(2), *V* = 2397.5(8) Å^3^, *F*(000) = 1380, μ = 3.584 mm^–1^, *D*_c_ = 1.916 g·cm^–3^, crystal size = 0.413 × 0.118 × 0.084 mm, θ = 3.2–28.9°, index range −13 ≤ *h* ≤ 17, −14 ≤ *k* ≤ 8, −23 ≤ *l* ≤ 22. Final *R* indices: *R*_int_ = 0.032, *R*[*F* ^2^ > 2σ­(*F* ^2^)] = 0.029, w*R*(*F* ^2^) = 0.066, *S* = 1.06. Largest diffraction peak and hole: 1.47 and −1.01 e·Å^–3^ (Table S1).^42^

C_10_H_20_ErFK_2_N_2_O_12_ (CCDC 2542953; Er2F2), *M* = 624.74, triclinic, space group P1̅, *Z* = 2, *a* = 8.804(2), *b* = 9.330(2), *c* = 12.765(3) Å, α = 87.23(3)°, β = 82.81(2)°, γ = 65.42(2)(6)°, *V* = 946.0 ^4^ Å^3^, *F*(000) = 610, μ = 4.949 mm^–1^, *D*_c_ = 2.193 g·cm^–3^, crystal size = 0.075 × 0.056 × 0.018 mm, θ = 2.4–30.5°, index range −12 ≤ *h* ≤ 12, −13 ≤ *k* ≤ 13, −18 ≤ *l* ≤ 17. Final *R* indices: *R*_int_ = 0.032, *R*[*F* ^2^ > 2σ­(*F* ^2^)] = 0.028, w*R*(*F* ^2^) = 0.063, *S* = 1.01. Largest diffraction peak and hole: 1.22 and – 0.84 e·Å^–3^ (Table S1).

C_20_H_35_ClEr_2_K_5_N_4_O_23.50_ (CCDC 2542951, Er2OH2), *M* = 1272.99, monoclinic, space group *I*2/*m*, *Z* = 2, *a* = 8.785(2), *b* = 24.223(6), *c* = 9.050(3) Å, β = 97.06(3), *V* = 1911.2(9) Å^3^, *F*(000) = 1238, μ = 5.067 mm^–1^, *D*_c_ = 2.212 g·cm^–3^, crystal size = 0.324 × 0.042 × 0.028 mm, θ = 3.4–28.8°, index range −7 ≤ *h* ≤ 11, −15 ≤ *k* ≤ 30, – 12 ≤ *l* ≤ 10. Final *R* indices: *R*_int_ = 0.023, *R*[*F* ^2^ > 2σ­(*F* ^2^)] = 0.019, w*R*(*F* ^2^) = 0.044, *S* = 1.08. Largest diffraction peak and hole: 1.03 and −0.90 e·Å^–3^ (Table S1).

C_2_H_12_N_6_O_4_S (CCDC 2535111), *M* = 602.67, cubic, space group *P*4332, *Z* = 2, *a* = 17.797(4) Å, *V* = 5637(4) Å^3^, *F*(000) = 2280, μ = 2.63 mm^–1^, *D*_c_ = 1.274 g·cm^–3^, crystal size = 0.32 × 0.24 × 0.10 mm, θ = 3.5–73.3°, index range −22 ≤ *h* ≤ 21, −21 ≤ *k* ≤ 21, −19 ≤ *l* ≤ 21. Final *R* indices: *R*_int_ = 0.018, *R*[*F*2 > 2σ­(*F*2)] = 0.020, w*R*(*F*2) = 0.057, *S* = 1.15. Largest diffraction peak and hole: 0.14 and −0.23 e·Å^–3^ (Table S1).^43^

## Powder Diffractograms

Powder diffractograms of guanidinium sulfate were recorded using a D8 ADVANCE diffractometer with a Cu lamp and Vantec detector (Figure S7).

## Spectroscopic Measurements

Electronic absorption spectra were recorded using a Cary 5000 UV–vis–NIR spectrophotometer at room temperature. During the titration of [Er­(EDTA)­(H_2_O)_2_]^−^ with all hydroxide solutions, the pH was measured with a Mettler Toledo Micro-Pro-IS 3 M KCl electrode.

The spectra of crystals were measured at two different positions with respect to the light ray at RT. The average spectra were obtained using eq ([Disp-formula eq4]).

4 $$\varepsilon_{\mathrm{av}}=\frac{\varepsilon_{I}+\varepsilon_{\mathrm{II}}}{2}$$

The average spectra were used for the calculation of the experimental oscillator strengths (*P*_exp_) of the observed bands using eq ([Disp-formula eq5])

5 $$P_{\exp}=\frac{4.32\times 10^{-9}}{c\cdot d}\times \int_{{\mathrm{\nu ̅}}_{1}}^{{\mathrm{\nu ̅}}_{2}}A\left(\mathrm{\nu ̅}\right)d\mathrm{\nu ̅}$$

where c is the concentration of Er^3+^ (mol·dm^–3^), *d* is the length of the optical path (cm), and *A*(*ν̅*) is the absorbance as a function of the *ν̅* wavenumber (cm^–1^).

Experimental oscillator strengths were used to calculate the phenomenological parameters Ω_λ_ (λ = 2, 4, 6) according to the Judd–Ofelt theory^44,45^ using eq ([Disp-formula eq6])

6 $$P_{\mathrm{cal}}=\chi \cdot \frac{8\pi^{2}\mathrm{mc}\mathrm{\nu ̅}}{3h\left(2J+1\right)}\cdot \sum_{\lambda =2,4,6}\Omega_{\lambda }{\left|\left\langle \psi^{\prime }J^{\prime }∥U^{\left(\lambda \right)}∥\psi J\right\rangle \right|}^{2}$$

where *P*_cal_ is the theoretical oscillator strength of the electric dipole transition, $\chi =\frac{{{(n}^{2}+2)}^{2}}{9n}$ (*n* is the refractive index, equal to 1.5 for crystals and 1.33 for solutions), m is the electron mass, *c* is the speed of light, *ν̅* is the band maximum wavenumber (cm^–1^), *h* is the Planck’s constant, and *J* is the ground-state quantum number of the lanthanide ion, and the reduced matrix elements of the respective unit tensor operator *U*(λ) are given by ⟨ψ^′^J′∥U^(λ)^∥ψJ⟩. The values of the |⟨ψ′J′∥U^(λ)^∥ψJ⟩|^2^ = U^(λ)^ operator were obtained from Carnall et al.^46^

The root mean square deviation (RMS) was used to determine the accuracy of the intensity parameter calculation using the following equation

7 $$\mathrm{rms}=\sqrt{\sum_{i=4}^{n}\frac{{\left(P_{\exp}^{\left(i\right)}-P_{\mathrm{cal}}^{\left(i\right)}\right)}^{2}}{n-m}}$$

where *P*_exp_ and *P*_cal_ are defined by eqs 5 and 6, respectively, *n* is the number of observed absorption bands, and *m* = 3 is the number of calculated parameters (Ω_2_, Ω_4_, Ω_6_).

## NMR Spectroscopy

^1^H NMR and ^89^Y NMR spectra were recorded using a Jeol 500 MHz NMR spectrometer. The ^89^Y chemical shifts were obtained using gradient-enhanced Heteronuclear Multiple Bond Correlation ^1^H-^89^Y (HMBC) experiments using a Jeol JNM-ECZ500R 500 MHz spectrometer equipped with a 5 mm ^1^H/^19^F/^2^H/^31^P-^109^Ag probe with a low-frequency unit with a delay for the evolution of long-range couplings of 62.5 ms and a 1.5 s delay for the relaxation time, according to Jeol’s pulse program (hmbc_pfg.jxp). For example, in an HMBC experiment, the raw data set consisted of 129 (F2) × 1739 (F1) complex data points, with spectral widths of 15 ppm (offset 5 ppm) and 400 ppm (offset 150 ppm) in the F1 and F2 dimensions, respectively.

## Pehametric Measurements

Pehametric measurements were carried out using Mettler Toledo Micro-Pro-IS 3 M KCl electrode. For calibration of the pH electrode, the following solutions were used: pH = 3 ± 0,05 HCl standard (Eurochem BGD); phosphate buffer pH = 6.88; bicarbonate buffer pH = 10; saturated at 25 °C Ca­(OH)_2_ solution pH = 12.45.^47,48^

## Osmometric Measurements

The osmolality of the solutions was measured using an Osmomat 030 Gonotec osmometer. For the calibration of the osmometer, the solutions listed in Table 2 were prepared.

**Table 2:** Calibration Solutions for Osmometric Measurements

Osmolality mOsm/kg H_2_O	NaCl g/100g H_2_O
100	0.3088
200	0.6260
300	0.9463
400	1.2685
500	1.5916
600	1.9148
700	2.2380
750	2.3980
850	2.7178
1200	3.8370
1800	5.7560
2000	6.3960
2500	7.9970

## EDS Spectra

The EDS spectra of **Er2OH2** crystals were measured using a scanning electron microscope (Hitachi S-3400N) with an EDS detector (Thermo Scientific Ultradry) to confirm the presence of Cl^–^ anions (Figure S8).

## ICP OES

The concentration of Er­(III) was determined by inductively coupled plasma atomic emission spectroscopy using a spectrometer (ARL model 3410 ICP OES). We used Fluka erbium standard solutions to determine the Er­(III) concentration.

## Calculations

Linear fitting was performed by using Microsoft Excel and OriginLab 16 software. Factor analysis was performed according to the procedure described in the literature.^49^

## Results and Discussion

### Crystal Structure

The crystallization of monomeric terminal hydroxo complexes of Ln­(III) cations is not straightforward. This can be attributed to the fact that the highly negative OH^–^ anion, being a strong Pearson base, has a high affinity for binding to hard acids such as 4*f*/5f elements. A critical perusal of the crystal data available in the CCDC database reveals that only dimeric and polymeric μ_2_,μ_3_-hydroxo complexes have been isolated in the solid state. Monomeric species with a terminal hydroxo anion have been established in only a few crystal structures involving bulky ligands, which prevent the formation of μ_2_- and μ_3_-hydroxo compounds.^50,51^ To overcome this problem, we decided to use a fluoride (F^–^) anion as a mimetic ligand for the hydroxide (OH^–^) anion. Both ligands are isoelectronic and exhibit similar, although not identical, coordination properties toward Ln­(III) cations. The use of fluoride ions to suppress the formation of hydroxo-bridged and polymeric lanthanide species is well precedented in the literature and is rationalized by the strong competitive binding of F^–^ and OH^–^ to Ln­(III), which have comparable thermodynamic stability.^52,53^ This competition has been discussed in terms of ligand exchange equilibria (e.g., LnF + OH^–^ ⇌ LnOH + F^–^),^54^ as well as the formation of structurally related species.^55,56^

The following compounds were isolated: Na­[Er­(EDTA)­(H_2_O)_3_]·3.5H_2_O (hereinafter referred to **ErW3**); K­[Er­(EDTA)­(H_2_O)_2_]·4H_2_O (**ErW2**); K_5_[Er­(EDTA)­(μ-OH)]_2_Cl·5.5H_2_O (**Er2OH2**); [C­(NH_2_)_3_]_2_[Er­(EDTA)­(F)­(H_2_O)]·2H_2_O (**ErWF**); [C­(NH_2_)_3_]_3_[Er­(EDTA)­F_2_]·H_2_O (**ErF2**); K_4_[Er­(EDTA)­(μ-F)]_2_·8H_2_O (**Er2F2**) and their crystal structure were determined. The Er–L bond lengths are collected in Table 3 and Table S2, and the crystallographic parameters are presented in Table S1 in Electronic Supplementary Data. The molecular structures of the complex anions are presented in Figure 1.

**Figure 1:**
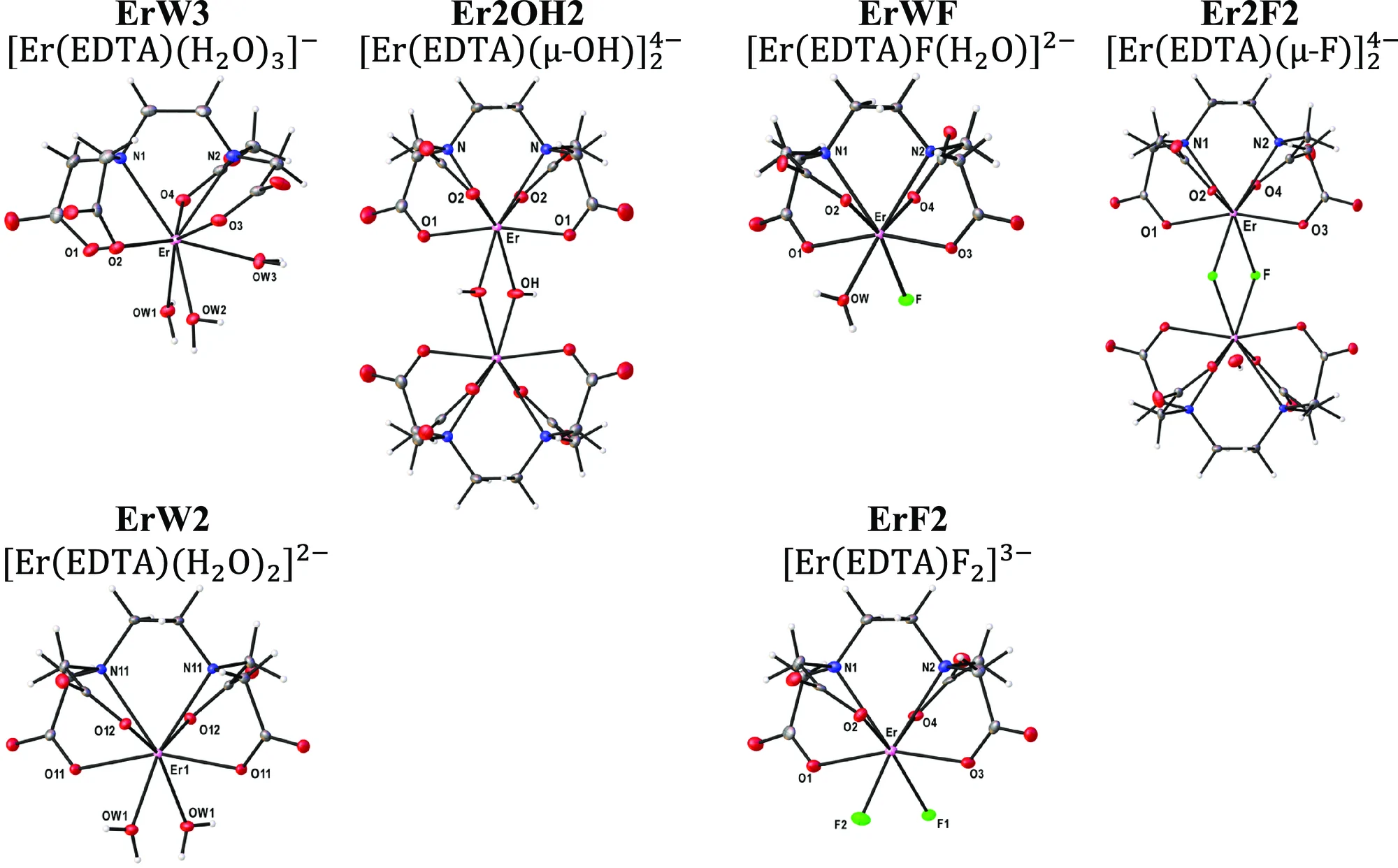
Molecular structures of the complex anions in the crystals under study.

**Table 3:** Selected Bond Length­(Å) Determined for **ErW3, ErW2, Er2OH2,ErWF, ErF2, and Er2F2**

	**Er–N**	**Er–O**_**carbox**_	**Er–H**_**2**_ **O**	**Er–OH**	**Er–F**
**ErW3**	2.587(5)–2.672(6)	2.345(4)–2.377(4)	2.340(4)–2.466(3)		
**ErW2**	2.557(2)–2.570(2)	2.280(2)–2.313(2)	2.346(2)–2.336(2)		
**Er2OH2**	2.673(2)	2.311(2)–2.331(2)		2.279(2)	
**ErWF**	2.609(2)–2.620(2)	2.297(2)–2.322(2)	2.352(2)		2.167(2)
**ErF2**	2.589(3)–2.634(3)	2.318(2)–2.334(2)			2.176(2)–2.208(2)
**Er2F2**	2.592(3)–2.605(3)	2.271(2)–2.303(2)			2.176(2)–2.288(2)

In all compounds, the EDTA ligand is coordinated in the same manner, namely, via four oxygen and two nitrogen atoms. The conformation of the EDTA ligand remains essentially unchanged in the 8-coordinate complex anions, whereas it is clearly different in the 9-coordinate compound in the **ErW3** crystal. Variations in the Er­(III) coordination number lead to changes in the conformation of the EDTA ligand and elongation of the Er­(III)–donor atom bond lengths, as described previously.^57^ Table 3 and Table S2 reveals a diversity of coligand interactions with the Er­(III) cation. The Er–coligand bond lengths follow the order: Er–(OH_2_) > Er–(μ-F^–^) ≈ Er–(μ–OH^–^) > Er–F^–^(terminal). The Er–F bond in monofluoride complex [Er­(EDTA)­F­(H_2_O)]^2–^ is the shortest among all the Er–coligand bonds considered. The replacement of a water molecule in the [Er­(EDTA)­F­(H_2_O)]^2–^ complex (**ErWF**) by the F^–^ ligand results in the formation of the difluoride [Er­(EDTA)­F_2_]^3–^ anion (**ErF2**), in which the Er–F1 and Er–F2 bond lengths differ by about 0.032 Å. The first fluoride coordination to [Er­(EDTA)­(H_2_O)_2_]^−^ is favored in solution, as indicated by the stability constant β_Er(EDTA)F_ = 65 ± 16 for the formation of [Er­(EDTA)­F­(H_2_O)]^2–^. In contrast, the second fluoride binding step, leading to [Er­(EDTA)­F_2_]^3–^, is significantly less favorable, as reflected by the much lower stability constant β_Er(EDTA)F_2__ = 2.^58^

This stepwise difference correlates with the structural data. The Er–F bond length in the monofluoride complex is 2.167(2) Å, whereas the difluoride complex exhibits two nonequivalent and generally longer Er–F distances (2.176(2) and 2.208(2) Å), indicating a change in the coordination environment upon coordination of the second fluoride ligand.

In the dimeric [Er­(EDTA)­μ_2_F]_2_^4–^ (**Er2F2**) form, the F^–^···F^–^ distance (2.537(4) Å) as well as the F–Er–F angle (67.6(1)°) are substantially smaller than those in the corresponding monomeric difluoride [Er­(EDTA)­F_2_]^3–^ anion (F^–^···F^–^ = 2.768 Å; < F1–Er–F2 = 78.3(1)°) in **ErF2**. On the other hand, it should be noted that the Er–F bonds are significantly shorter in the monomeric complexes. A clear structural similarity is seen between the dimeric [Er­(EDTA)­(μ-F)]_2_^4–^ species in **Er2F2** (*C*_*2*_ symmetry) and the dimeric [Er­(EDTA)­(μ-OH)]_2_^4–^ species in **Er2OH2** (C_2h_ symmetry). Both Er–OH bonds are equivalent (2.279(2) Å), while Er–F1 is about 0.112 Å longer than Er–F2. In [Er­(EDTA)­(μ-F)]_2_^4–^ (**Er2F2**), the Er–F1 bond length (2.288(2) Å) is comparable, while the Er–F2 bond (2.176(2) Å) is markedly shorter than the Er–OH bond (2.279(2) Å) in [Er­(EDTA)­(μ-OH)]_2_^4–^ (**Er2OH2**). Nevertheless, the OH^–^ anion exerts a stronger perturbing effect on the interaction between Er­(III) and the chelating EDTA ligand, as indicated by the elongation of the Er–O (carboxylate) and Er–N bonds. In the [Er­(EDTA)­(μ-OH)]_2_^4–^ dimer (**Er2OH2**), these bonds are, on average, 0.031 Å and 0.071 Å longer, respectively, than in the corresponding [Er­(EDTA)­(μ-F)]_2_^4–^ (**Er2F2**) complex anion. Thus, the Er­(III) cation is less tightly wrapped by the chelating EDTA ligand because the OH^–^ anions pull the Er­(III) cation out of the EDTA cage. A similar Er­(III) pulling-out effect induced by the F^–^ coligand was observed in the monomeric difluoride complex anion in the **ErF2** crystal, although to a slightly lesser extent. Figure 2 illustrates the average Er–L bond lengths in relation to the Er­(III) coordination number and coordinated coligands (H_2_O, OH^–^, F^–^).

**Figure 2:**
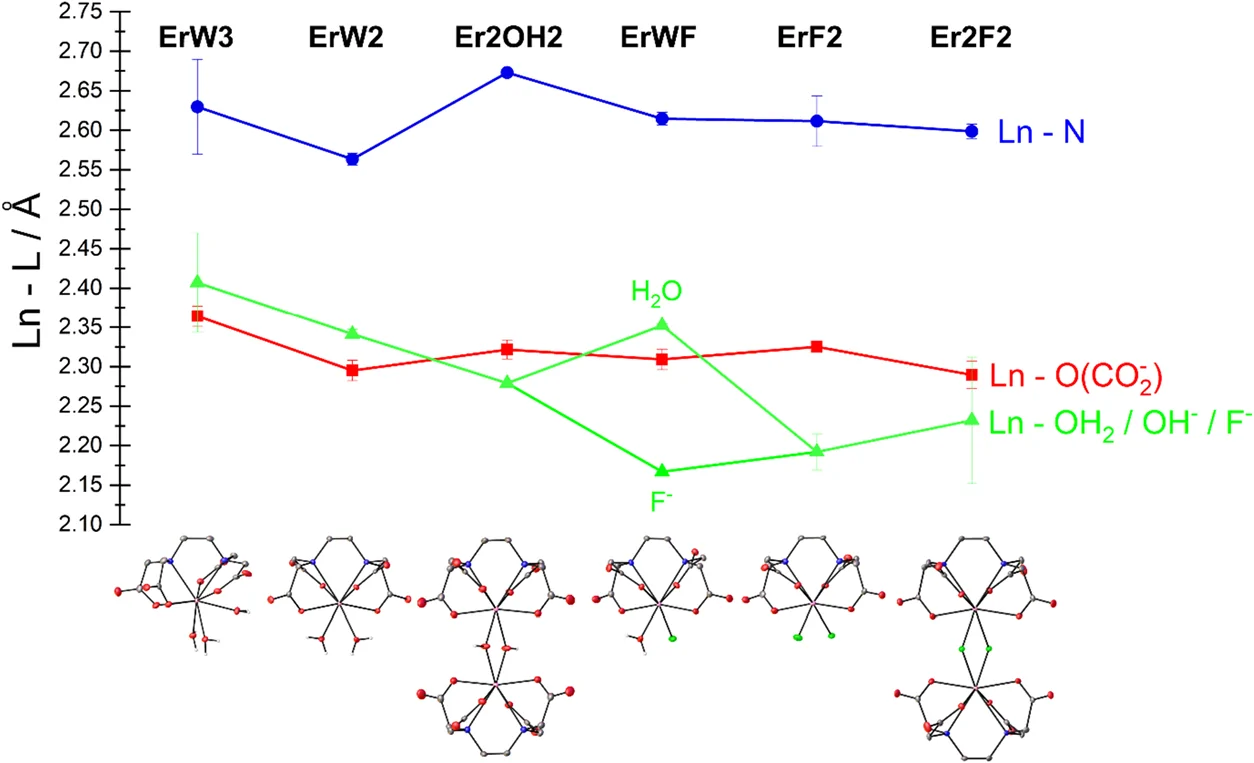
Average Er–L bond lengths in **ErW3**, **ErW2**, **Er2OH2, ErWF, ErF2, and Er2F2** vs the Er­(III) coordination number and the type of coordinated coligands (H_2_O, OH^–^, F^–^).

As can be seen in the fluoride complexes under study, the Er­(III) ion is more tightly encapsulated within the resulting ternary complex, while the OH^–^ ligands lead to weakening the Er–N and Er–O bonds compared with the F^–^ ligands.

This effect is further reinforced by the outer-sphere interactions between the potassium cations and the OH^–^ and F^–^ ligands, as shown in Figure 3.

**Figure 3:**
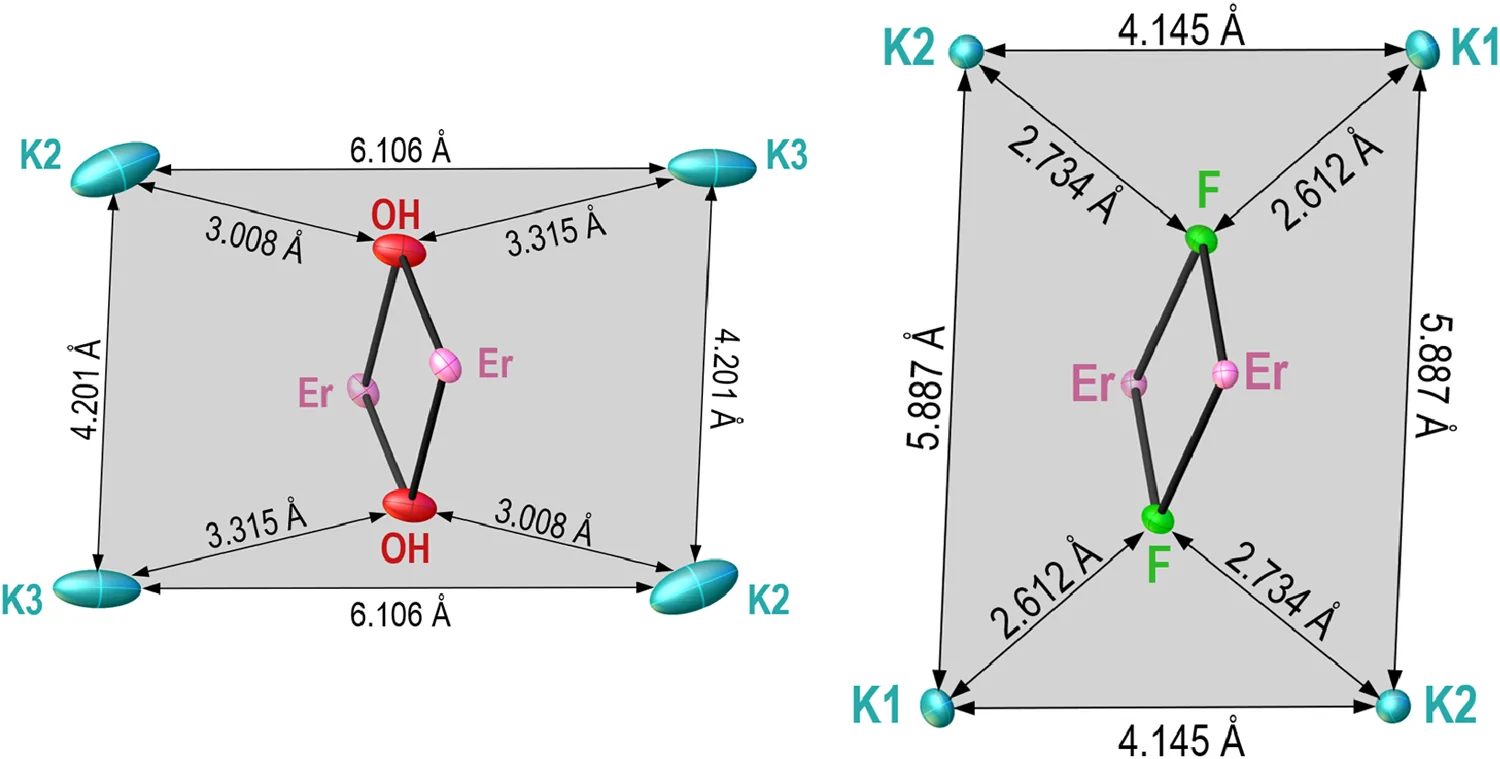
K_4_(OH)_2_ and K_4_F_2_ planes in the K_5_[Er­(EDTA)­(μ-OH)]_2_Cl·5.5H_2_O (left) and K_4_[Er­(EDTA)­(μ-F)]_2_·8H_2_O (right) crystals, respectively.

Four potassium cations and two OH^–^/F^–^ anions lie in the same plane in the respective crystals, with the K^+^···OH^–^ distance being, on average, 0.50 Å longer than K^+^···F^–^. The hydrogen atom of the hydroxide anion repels the potassium cations, thereby weakening the K^+^···OH^–^ interaction. Taking into account the aforementioned data, it is likely that the hydroxy [Er­(EDTA)­(OH)­(H_2_O)]^2–^ complex is more stable than the fluoride analogue. The F^–^ and OH^–^ anions interact with the out-of-sphere K^+^ cations, which partially shield them from direct interactions with water molecules. A possible explanation is the contribution of the covalent character to the Er–ligand bonding. As previously demonstrated for the Gd­(III) analogues, the degree of covalency is not linearly related to the bond length.^59^ For very short Er–ligand bonds, such as the Er–OH and Er–F bonds observed here, covalent effects may become significant and influence the overall stability and structural properties of the complexes. Therefore, despite the weaker K^+^···OH^–^ interaction compared with K^+^···F^–^, the stronger Er–OH bonding may provide additional stabilization of the hydroxo complex and contribute to the observed structural differences.

A slightly different situation is observed for other monomeric fluoride compounds (**ErWF** and **ErF2**), in which the fluoride anions interact with both water molecules and guanidinium cations via hydrogen bonds (see Table S3a-f in the ESI). A similarly strong interaction is observed between the guanidinium cation and the oxygen atoms of the carboxylate groups, as well as those of the water molecules.^19,60^

The observed differences between the K^+^ and [C­(NH_2_)_3_]^+^ cations in their interactions with OH^–^/F^–^ anions may indicate a distinct influence of these cations on the formation and stability of the ternary [Er­(EDTA)­(OH)­(H_2_O)]^2–^ complex in solution.

It should be noted that the difference in the interaction strengths of K^+^ cations with [Er­(EDTA)­(μ-OH)]_2_^4–^ and [Er­(EDTA)­(H_2_O)_2_]^−^ complex anions is also reflected in the shortest K···Er distances in the respective crystal structure, which are approximately ∼4.1 Å in **Er2OH2** and ∼5.7 Å in **ErW2**. The question arises as to whether this property is connected to the stability of the complex anions in solution. Therefore, in the next step, the effect of the ionic medium on the stability of the ternary complexes in aqueous solution was investigated.

### Hydroxy Species in Solution: Stoichiometry and the Coordination Number of Er­(III)

To elucidate the stoichiometry of the [Er­(EDTA)­(OH)_*x*_(H_2_O)_*y*_]^(x+1)–^ species and the coordination number of Er­(III) cation, in strongly basic solutions, UV–vis–NIR electronic absorption spectroscopy and ^89^Y NMR spectroscopy were employed. The hypersensitive ^4^I_15/2_ → ^4^G_11/2_, ^4^I_15/2_ → ^2^H_11/2_ transitions, together with the ^4^I_15/2_ → ^4^S_3/2_ transition, are presented in Figure 4.

**Figure 4:**
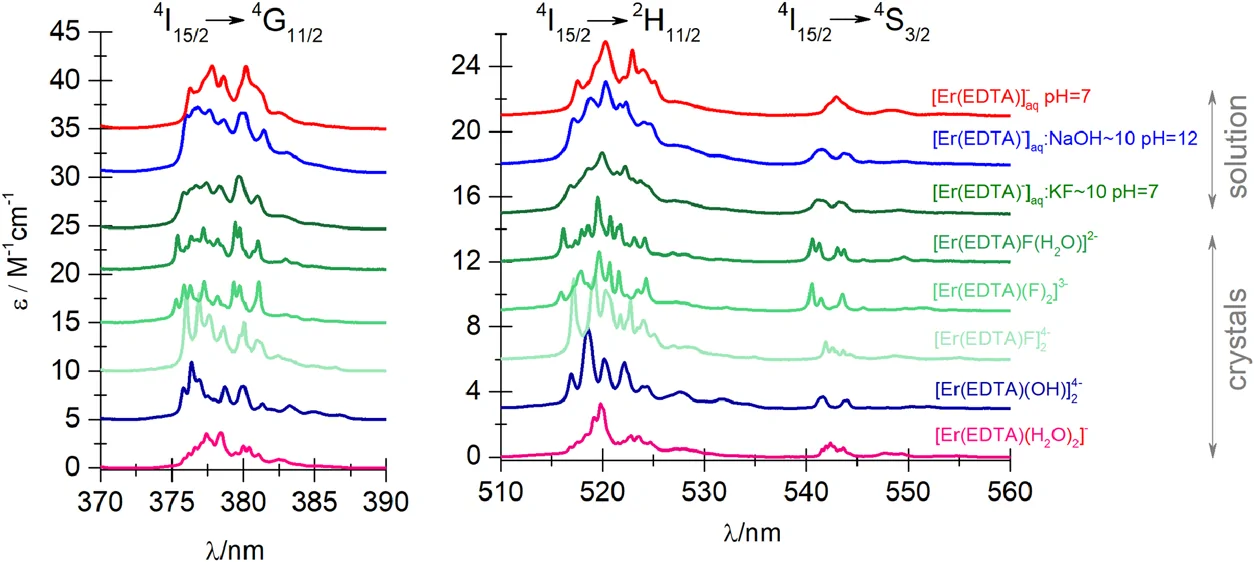
Selected f–f transitions of the Er­(III) systems under study.

The oscillator strengths of the f–f transitions recorded in the 350–1100 nm spectral range for the Er­(III) systems under study are listed in Table S4. To ensure comparable experimental conditions, the [Er­(EDTA)­(H_2_O)_*x*_]:NaOH (hereinafter referred to as **ErOH­(S)**) and [Er­(EDTA)­(H_2_O)_*x*_]:KF (hereinafter referred to as **ErF­(S)**) molar ratios in the respective aqueous solutions were adjusted to approximately 1:10. According to Király et al., under these conditions, the monofluoride [Er­(EDTA)­F­(H_2_O)]^2–^ species predominates in aqueous solutions.^58^ A close similarity in the spectroscopic features, including both the intensities and the spectral pattern of the f–f bands, is observed among the aforementioned solutions, **ErWF**, and **ErF2** monocrystals.

Although the ^4^I_15/2_ → ^4^S_3/2_ transition is not classified as a hypersensitive transition, it is a valuable structural probe owing to the low J value of the ^4^S_3/2_ excited state. The relatively simple Stark splitting pattern associated with this transition facilitates the identification of spectral changes arising from variations in the local coordination environment of the Er­(III) ion, particularly changes in the coordination number and symmetry, as previously demonstrated. However, since both the ground and excited states are split by the crystal field, structural interpretations based solely on this transition should be made with caution.^29,57^

Interestingly, the f–f transition spectra of **ErWF** and **ErF2** are significantly more similar to each other than to those of the dimeric complexes **Er2F2** and **Er2OH2**. At the same time, the spectra of **Er2F2** and **Er2OH2** display certain similarities. This confirms that the fluoride anion may, to some extent, mimic the coordination properties of the hydroxide anion. These results suggest that the hydroxo species present in the basic aqueous solution is an eight-coordinate monohydroxide complex with the formula [Er­(EDTA)­(OH)­(H_2_O)_*x*_]^2–^. However, it should be noted that direct comparisons of the spectroscopic properties of crystals and solutions, particularly for dynamic systems such as [Er­(EDTA)­(H_2_O)_*x*_]^−^ complexes, must be interpreted with caution. Nevertheless, this approach provides valuable insights into the structure and coordination environment of the species present in the solution. Consequently, although indirect, this methodology provides a valuable tool for probing the solution structures of lanthanide complexes (see Figure S9 and the accompanying discussion in the ESI).

It is worth examining the Judd–Ofelt Ω_λ_ parameter values in more detail (Table S4), as these parameters are a “fingerprint” of a given system and describe the intensities of all f–f transitions present in the Er­(III) spectrum. Since the U(2) values used in eq ([Disp-formula eq6]) are highest for hypersensitive ^4^I_15/2_ → ^4^G_11/2_ and ^4^I_15/2_ → ^2^H_11/2_ transitions, the Ω_2_ parameter is mainly influenced by the intensities of these bands. Among the Ω_λ_ parameters, Ω_2_ is sensitive to the polarizability of coordinated ligands,^61,62^ covalent interactions,^63^ and vibronic effects,^64^ whereas Ω_4_ and Ω_6_ are primarily governed by covalent interactions and crystal-field effects.^65^ In general, it is difficult to identify which of these mechanisms has the dominant influence on the Ω_2_ values, as the Judd–Ofelt parameters reflect the combined perturbing effects of all these physical contributions.^66^ In the compounds under study, the situation is relatively simple, as the coordination sphere of Er­(III) in all crystals is predominantly occupied by six donor atoms of the EDTA ligand, while the two remaining coordination sites are occupied by H_2_O, OH^–^, or F^–^, as well as in previously published [C­(NH_2_)_3_]_3_[Er­(EDTA)­(CO_3_)]·H_2_O crystal by the CO_3_^2–^ ligand. In this way, the geometry of the coordination complex remains relatively unchanged, while the observed differences in the spectroscopic properties of the f–f transitions are mainly caused by the coordinated coligands.

The coligand polarizabilities (α), Er–coligand bond lengths (*r*), Ω_2_ parameter values, as well as the $(\sum {\frac{\alpha }{r^{4}})}^{2}$ factor, are presented in Table S5. A strong correlation was found between the Ω_2_ values and the $(\sum {\frac{\alpha }{r^{4}})}^{2}$ factor, with a Pearson correlation coefficient of 0.92. This result indicates that ligand polarizability is one of the main factors affecting the intensities of f–f transitions, particularly hypersensitive transitions, which are well represented by the Ω_2_ parameter.

A similar dependence is observed for aqueous solutions of the [Er­(EDTA)­(F)­(H_2_O)]^2–^ and [Er­(EDTA)­(OH)­(H_2_O)]^2–^ complexes, for which the Ω_2_ parameter is larger for the hydroxy complex. However, when comparing the Ω_2_ values of the dimeric **Er2OH2** and **Er2F2** compounds, the Ω_2_ parameter is lower for the hydroxo-containing species than for the fluoride analogue, despite the greater polarizability of the OH^–^ ligand. In particular, the higher symmetry of the **Er2OH2** dimer (C_2h_) compared to that of **Er2F2** (C_2_) leads to a decrease in the Ω_2_ parameter. This observation indicates that molecular symmetry also plays an important role in governing Ω_2_ values.

Finally, ^89^Y NMR spectra were recorded at different pH values in order to assess the effect of hydrolysis on the chemical shift and coordination number of the yttrium cation. Y­(III) complexes are commonly used as structural analogues of heavier lanthanide­(III) ions, particularly Dy­(III), Ho­(III), and Er­(III), owing to their comparable ionic radii. The ^89^Y NMR spectra are presented in Figure 5.

**Figure 5:**
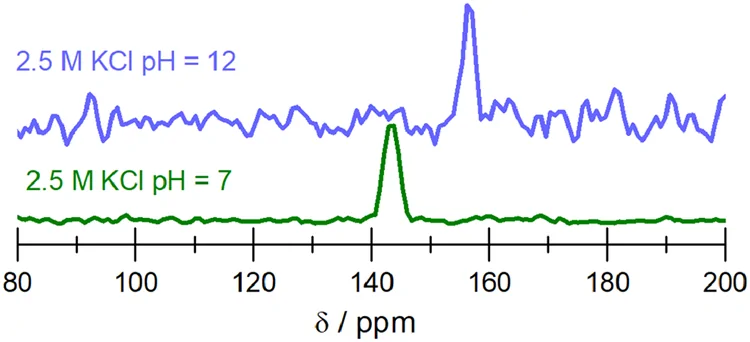
^89^Y NMR spectra of aqueous (D_2_O) solutions of the Y­(III)–EDTA system at different pH values.

According to empirical formulas^67^ the ^89^Y chemical shift of polyaminopolycarboxylates is linearly correlated with the number of donor atoms of each class. The experimental value of the ^89^Y NMR chemical shift of the Y­(III)–EDTA complex in solution (143.4 ppm) is close to that predicted from the empirical relation (134 ppm). In a more basic solution (pH ∼ 12), the ^89^Y NMR signal is shifted slightly downfield to 156.25 ppm. Assuming that the monohydroxide species predominates under these conditions, the shielding contribution of the OH^–^ anion was estimated by considering both the eight-coordinate species (with one water molecule [Y­(EDTA)­(OH)­(H_2_O)]^2–^) and the nine-coordinate species (with two water molecules [Y­(EDTA)­(OH)­(H_2_O)_2_]^2–^). Accordingly, the following values of S_OH_ were obtained: 89.2 for the 8-coordinate species and −18.9 for the 9-coordinate species. In comparison with the other shielding S_*x*_ parameters (which for the other O-donor atoms range from 89.5 for amide oxygen to 107.6 for water molecules), the value of 89.2 appears to be more reliable. Moreover, a slight change in the ^89^Y NMR chemical shift was observed for the 2.5 M KCl and 2.0 M NaCl solutions (156.25 and 154.69 ppm, respectively). The downfield shift in the KCl solution may indicate subtle differences between the interactions of K^+^ and Na^+^ ions with the [Y­(EDTA)­(OH)­(H2O)]^2–^ complex.

In summary, the presented UV–vis-NIR spectroscopic properties of the solid states and solutions together with ^89^Y NMR data indicate that the 8-coordinate [Er­(EDTA)­(OH)­(H_2_O)]^2–^ complex is the predominant species in basic aqueous solutions.

At this stage, the question arises regarding the influence of the medium effects and, ultimately, the thermodynamic stability of the [Er­(EDTA)­(OH)­(H_2_O)]^2–^ complex.

### Thermodynamic Investigation of the Hydrolysis Reaction

The spectroscopic and pH-metric titration of the Er­(III)-EDTA system was performed at different ionic media: NaCl, KCl, [C­(NH_2_)_3_]­Cl, and [N­(CH_3_)_4_]Cl at varying concentrations. Taking into account that both [Er­(EDTA)­(H_2_O)_2_]^−^ (see ref 57) and [Er­(EDTA)­(OH)­(H_2_O)]^2–^ complex anions are eight coordinate, the equilibrium constant β_OH_ of reaction ([Disp-formula eq8]) were determined at different conditions (0.1 M; 0.5 M; 1 M; 2 M; 3 M; 4 M) at ambient temperature.

8 $${[\mathrm{Er}\left(\mathrm{EDTA}\right)\left(H_{2}O{)}_{2}\right]}^{-}+OH^{-}⇄{\left[\mathrm{Er}\left(\mathrm{EDTA}\right)\left(\mathrm{OH}\right)\left(H_{2}O\right)\right]}^{2-}+H_{2}O$$

The influence of hydroxo-containing lanthanide species on anion binding has also been demonstrated for the [Er­(EDTA)­(CO_3_)]^3–^ and Ln–DO3A–X complexes (where DO3A = 1,4,7,10-tetraazacyclododecane-1,4,7-triacetate, X = O-donor coligands), where competitive coordination of hydroxide was shown to reduce the apparent affinity toward other anionic coligands.^29,68^

To exclude the formation of polymeric hydroxo species, an additional experiment was performed. The equilibrium constant was determined at three different total ErEDTA concentrations (1.55, 3.86, and 7.73 mM) and at a constant ionic strength (0.1 M NaCl) (Figure S1). The obtained values of the equilibrium constant of reaction 8 remained constant within the experimental error (logβ_OH_ = 2.02 ± 0.08 for c_ErEDTA_ = 1.55 mM, 1.96 ± 0.07 for c_ErEDTA_ = 3.86 mM, 2.13 ± 0.06 for c_ErEDTA_ = 7.73 mM), indicating that the hydrolyzed species is monomeric and that no concentration-dependent polymerization occurs.

The ionic product of water in various electrolytes was obtained from the literature,^69^ except for that in the [C­(NH_2_)_3_]Cl medium. In this case, the arithmetic average of the corresponding p*K*_w_ values for the NaNO_3_ and KNO_3_ solutions was used.^69^ This assumption may, at least in part, be related to similar values of logγ_±_ for [C­(NH_2_)_3_]­Cl^70^ and KNO_3_ ^71^ as well as the structural analogy between the guanidinium-nitrate and potassium-chloride ion pairs. The selected spectra of the systems under study in various pH and ionic media are presented in Figure 6.

**Figure 6:**
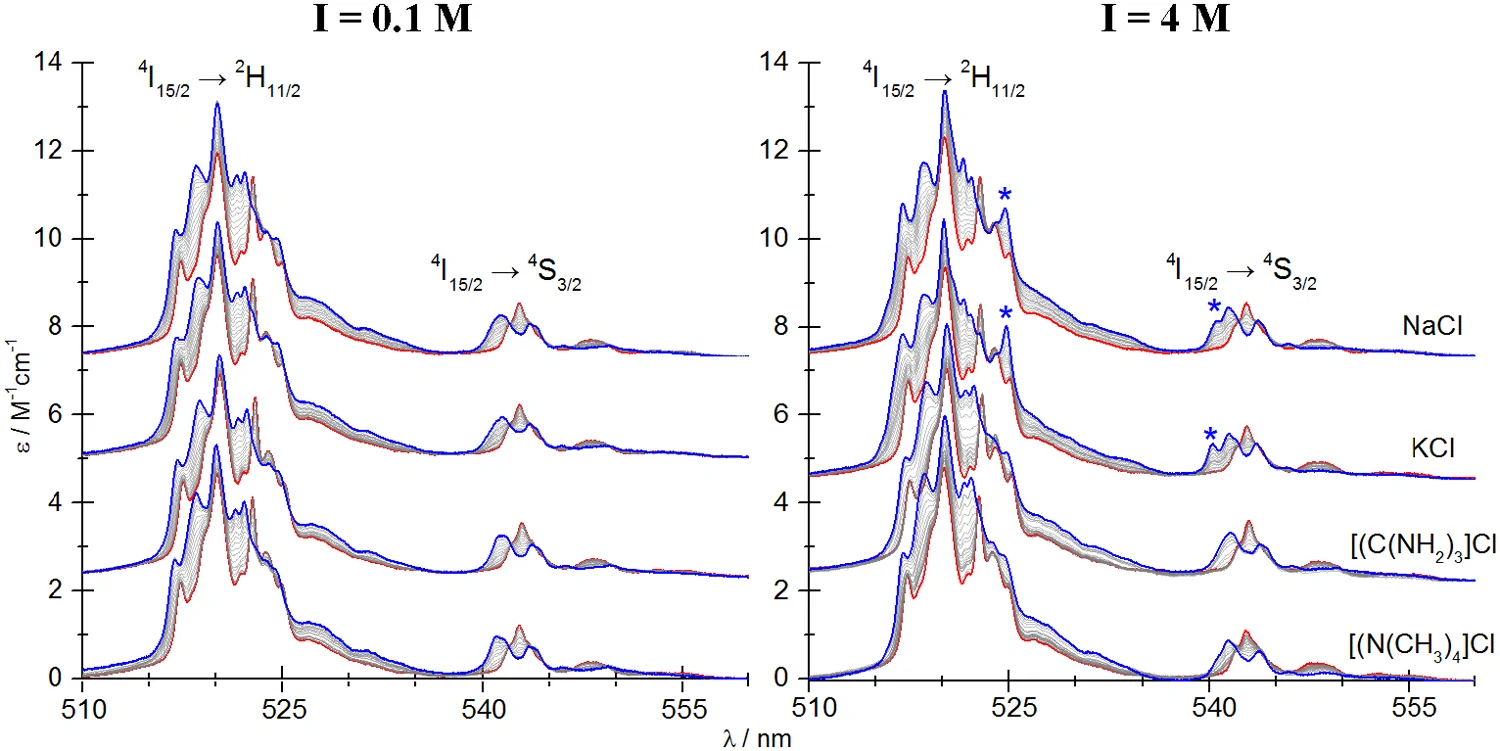
Absorption spectra of the ^4^I_15/2_ → ^2^H_11/2_ and ^4^I_15/2_ → ^4^S_3/2_ transitions of the Er­(III)–EDTA system recorded at different pH values between ∼7 (spectra marked in red) and 12.5 (spectra marked in blue) in various ionic media. Additional bands observed in the spectra of the KCl and NaCl solutions were labeled with asterisks.

The initial spectra (highlighted in red) of the ^4^I_15/2_ → ^2^H_11/2_ and ^4^I_15/2_ → ^4^S_3/2_ transitions at various ionic strengths are very similar, regardless of the electrolyte used, whereas the final spectra (marked in blue) for NaCl and, particularly, KCl solutions show significant differences. These results indicate that the stability of the hydroxy complex is likely modulated by the composition of the ionic medium. Factor analysis of the recorded spectra revealed that two main species, [Er­(EDTA)­(H_2_O)_2_]^−^ and [Er­(EDTA)­(OH)­(H_2_O)]^2–^, coexist in the solutions under study. Furthermore, the corresponding equilibrium constants were calculated using the determined molar fractions of these complexes. Next, the experimental values of logβ_OH_ were extrapolated to zero ionic strength using the SIT equation (eq (3)), and the results are presented in Figure 7.

**Figure 7:**
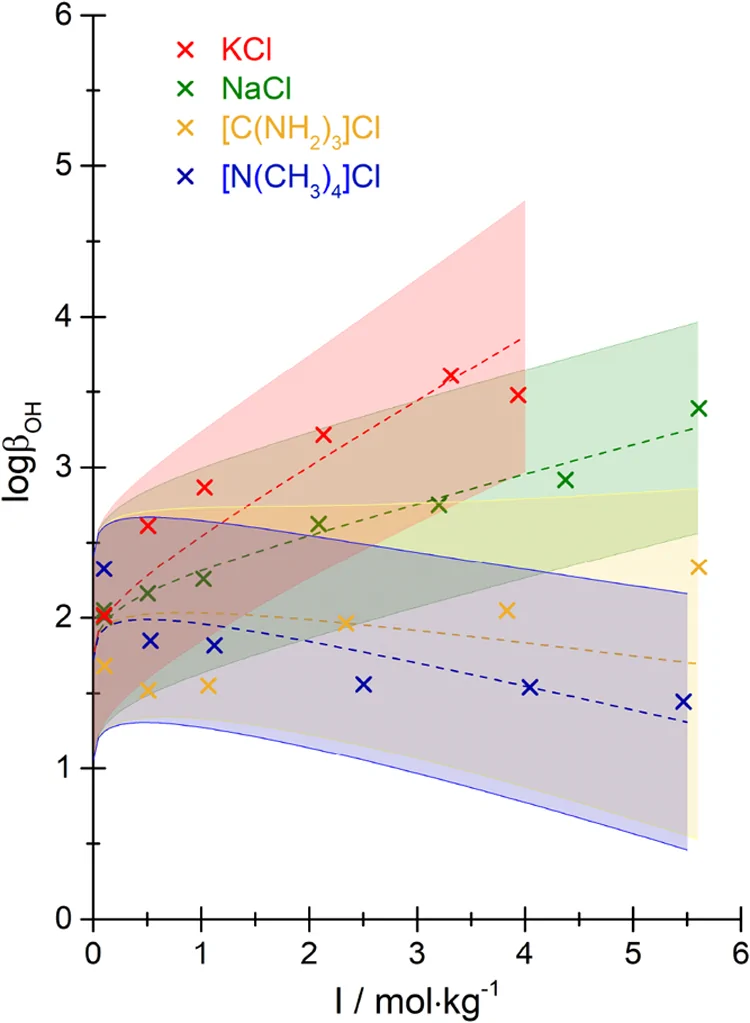
Extrapolation of experimental data to I = 0 for the formation of Er­[(EDTA)­(OH)­(H_2_O)]^2–^ in different electrolytes (KCl, NaCl, [C­(NH_2_)_3_]­Cl, and [N­(CH_3_)_4_]­Cl) using the specific ion interaction theory (SIT).

In the first step, the log β_OH_^o^ values were extrapolated using the SIT equation, treating each electrolyte independently. Simultaneously, the Δε values defined by eq ([Disp-formula eq9]) exhibited significant scatter; therefore, in the next step, the averaged value of log β_OH_^°^ = 1.73 ± 0.23 was taken as the reliable value, and the Δε values were recalculated. The values obtained in this way are presented in Table 4.

9 $$\Delta \varepsilon =\varepsilon_{M-\left[\mathrm{Er}\left(L\right)\right]}+\varepsilon_{\mathrm{OH}}-\varepsilon_{M-\left[\mathrm{Er}\left(L\right)\left(\mathrm{OH}\right)\right]}$$

**Table 4:** Calculated Values of log β_OH_^o^ and Δε for Systems under Study along with the Literature ε_X‑Cl_ Values and Partial Molal Volumes V^o^

electrolyte		Δε	ε_X‑Cl_	V° cm^3^·mol^–1^
NaCl	1.79 ± 0.03	0.15 ± 0.01		
KCl	2.02 ± 0.09	0.35 ± 0.07		
[C(NH_2_)_3_]Cl	1.32 ± 0.10	0.01 ± 0.06		
[N(CH_3_)_4_]Cl	1.78 ± 0.19	–0.25 ± 0.08		
**average**	**1.73 ± 0.23**			
NaCl	1.73 ± 0.23	0.18 ± 0.01	0.03 ± 0.01^35^	16.6 ^72^
KCl	1.73 ± 0.23	0.41 ± 0.05	0.0 ± 0.01^35^	26.8 ^72^
[C(NH_2_)_3_]Cl	1.73 ± 0.23	–0.10 ± 0.06	–0.08 ± 0.01^70^	69.6 ^73^
[N(CH_3_)_4_]Cl	1.73 ± 0.23	–0.17 ± 0.03	–0.09 ± 0.02 ^74^	107.4 ^72^

Figure 7 and Table 4 reveal that the log β_OH_ values increase with the ionic strength of the solution for the inorganic cations Na^+^ and K^+^. In contrast, for the larger organic cations, the log β_OH_ values are nearly independent of the ionic strength in the case of guanidinium cation and even exhibit a decreasing trend with increasing [N­(CH_3_)_4_]Cl concentration. Interestingly, the estimated Δε as well as ε_X‑Cl_ values^35,70,74^ are correlated with the partial molal volume of the electrolyte.^72,73^ In general, negative values of the interaction parameter ε_γ_ indicate favorable short-range interactions corresponding to stabilizing ion–ion interactions in solution. In contrast, positive values reflect repulsive or destabilizing interactions. Within the SIT approach, these parameters quantify specific deviations from the ideal behavior arising from short-range ion–ion interactions in electrolyte solutions.^75–77^

The strongest spectroscopic effect of the ionic medium is observed for the KCl electrolyte, whereas it is less pronounced for NaCl solutions. Therefore, gravimetric titrations of the basic solutions of Er­(III)–EDTA–OH were carried out. This experiment involved the addition of a small amount of solid supporting electrolyte (NaCl, KCl, and CsCl) to an aqueous solution of the Er­(III)–EDTA–OH complex. The binding constant of reaction (10)

10 $$M^{+}+{\left[\mathrm{Er}\left(\mathrm{EDTA}\right)\left(\mathrm{OH}\right)\left(H_{2}O\right)\right]}^{2-}⇌M{\left[\mathrm{Er}\left(\mathrm{EDTA}\right)\left(\mathrm{OH}\right)\left(H_{2}O\right)\right]}^{-}$$

are as follows: β_Na_ = 0.34 ± 0.09 (for NaCl), β_K_ = 0.6 ± 0.07 (for KCl), and β_Cs_ = 0.5 ± 0.1 (for CsCl), which are approximately 3 orders of magnitude smaller than β_OH_^o^ (see Table 4). The recorded spectra are presented in Figure S10. However, it should be noted that the errors in the estimated quantities are large because the experiments were carried out at varying ionic strengths. This data also indicates a slightly stronger interaction of K^+^ cations with the hydrolyzed [Er­(EDTA)­(OH)­(H_2_O)]^2–^ complex than Na^+^, which is consistent with the aforementioned conclusion that potassium cations stabilize the formation of the hydroxo complex to a greater extent than the other cations under study. This subtle binding property of the K^+^ cation likely arises from its spatial fit to oxygen donor atoms from the carboxylate and OH^–^ groups (the ionic radii of K^+^ and O^2–^ are almost identical). The differences in specific interactions between the K^+^–[Er­(EDTA)­(OH)­(H_2_O)]^2–^ and Na^+^–[Er­(EDTA)­(OH)­(H_2_O)]^2–^ ionic pairs should be reflected in their ε (SIT) parameters.

As no data on these parameters are available in the literature, osmometric measurements for the K^+^–[Er­(EDTA)­(H_2_O)_2_]^−^ and Na^+^–[Er­(EDTA)­(H_2_O)_2_]^−^ systems were first performed (Figure S12 and Table S6a,b), and the ε parameter values for K^+^–[Er­(EDTA)­(OH)­(H_2_O)]^2–^ and Na^+^–[Er­(EDTA)­(OH)­(H_2_O)]^2–^ were then estimated using eq ([Disp-formula eq9]).

Next, eq 11 was used to determine ε_γ_ = ln(10)·ε.

11 $$\phi_{0}-1=-\frac{\left|z_{+}z_{-}\right|\cdot A\gamma }{{\left(1,5\right)}^{3}\cdot I}\left(1+1,5\sqrt{I}-2\ln\left(1+1,5\sqrt{I}\right)-\frac{1}{1+1,5\sqrt{I}}\right)+\frac{v_{+}v_{-}}{v_{+}+v_{-}}\varepsilon_{\gamma }m$$

where ϕ_0_ is the osmotic coefficient, *z* ± is the ion charge, $A_{\gamma }=1.13\;kg^{1/2}\cdot {\mathrm{mol}}^{-1/2}$ (at 273 K, 1 bar), *I* is the ionic strength of the solution, *ε*_γ_ is the SIT interaction coefficient, and *m* is the molality of the solution.

The ε values determined were ε_Na–Er(EDTA)_ = −0.10 ± 0.02 and ε_K–Er(EDTA)_ = −0.13 ± 0.02. Unfortunately, similar experiments could not be performed for analogous guanidinium and tetramethylammonium compounds. According to Grenthe et al., specific ion interaction coefficients are available for many cations, and where experimental data are lacking, they can be estimated using correlations.^78^ Selected ε (SIT) parameters for aqueous solutions of sodium and potassium salts were determined and critically assessed.^35,78,79^ Only a few examples of activity and osmotic coefficients for guanidinium and tetramethylammonium salts have been reported in the literature (see references in Table 5). These data were used to estimate the ε parameter and are summarized in Table 5.

**Table 5:** Parameter ε for Na^+^, K^+^, [C­(NH_2_)_3_]^+^ and [N­(CH_3_)_4_]^+^ Compounds^a^

Anion	Na^+^	K^+^	[C(NH_2_)_3_]^+^	[N(CH_3_)_4_]^+^
HCO_3_^–^	–0.06 ± 0.01 ^80^	–0.07 ± 0.01 ^81^		0.05 ± 0.41 ^82^
SCN^–^	0.05 ± 0.01 ^35^	–0.01 ± 0.01 ^35^	–0.08 ± 0.05 ^83^	
F^–^	0.02 ± 0.02 ^35^	0.03 ± 0.03 ^35^	–0.05 ± 0.05 ^84^	0.20 ± 0.02 ^85^
NO_3_^–^	–0.04 ± 0.03 ^35^	–0.11 ± 0.04^35^	–0.14 ± 0.02 ^86^	–0.04 ± 0.08 ^86^
OH^–^	0.04 ± 0.01 ^35^	0.09 ± 0.01 ^35^	***–0.05 ± 0.05*** ^b^	0.35 ± 0.18 ^82^
Cl^–^	0.03 ± 0.01 ^35^	0.00 ± 0.02 ^35^	–0.07 ± 0.04 ^86^	–0.02 ± 0.06 ^85^
Br^–^	0.05 ± 0.01 ^35^	0.01 ± 0.02 ^35^	–0.06 ± 0.04 ^86^	–0.04 ± 0.07 ^85^
I^–^	0.08 ± 0.02 ^35^	0.02 ± 0.01 ^35^	–0.06 ± 0.03 ^86^	–0.32 ± 0.02 ^85^
ClO_4_^–^	0.01 ± 0.01 ^35^		–0.10 ± 0.04 ^86^	
CO_3_^2–^	–0.17 ± 0.07 ^87^	–0.09 ± 0.07 ^88^	–0.31 ± 0.13 ^83^	
SO_4_^2–^	–0.12 ± 0.07 ^35^	–0.06 ± 0.02 ^35^	–0.2 ± 0.05 ^89^	
CH_3_CO_2_^–^	0.06 ± 0.02 ^35^	0.09 ± 0.01 ^35^	–0.04 ± 0.02 ^83^	
C_2_H_5_CO_2_^–^	0.10 ± 0.01 ^90^	0.16 ± 0.11 ^91^	–0.05 ± 0.03 ^92^	
C_6_H_5_CO_2_^–^	0.01 ± 0.01 ^93^	0.02 ± 0.05 ^93^	–0.21 ± 0.03 ^92^	
[Er(L)]^−^	**–0.10 ± 0.02** ^b^	**–0.13 ± 0.04** ^b^	***–0.21 ± 0.04*** ^b^	***–0.28 ± 0.1*** b
[Er(L)(OH)]^2−^	**–0.24 ± 0.09** ^b^	**–0.45 ± 0.12** ^b^	***–0.16 ± 0.18*** ^b^	***0.2 ± 0.2*** ^b^

a The values marked in bold were determined, while the interpolated data (York regression, see below) are shown in italics.

b Determined in this paper.

Subsequently, linear relationships between ε_K+/Na+_ and ε_[C(NH2)3]+_ were established using the York regression method.^94,95^ This approach is particularly suitable for determining linear relationships between empirical parameters when uncertainties are present in both variables. For comparison, two additional approaches were applied, namely linear wage regression (hereinafter referred to as LWR) and regression without wage (RWW). The resulting relationships are presented in Figure 8 and Table 6.

**Table 6:** Parameters *a* and *b* Determined Using Different Methods: YR (York Regression), LWR (Weighted Linear Regression), RWW (Regression without Weights)

	ε_Na_ = **a**·ε_G_+ **b**		ε_K_ = **a**·ε_G_+ **b**	
	**a**	**b**	**a**	**b**
YR	1.13 ± 0.19	0.13 ± 0.02	1.07 ± 0.11	0.11 ± 0.03
LWR	1.28 ± 0.08	0.14 ± 0.02	0.78 ± 0.02	0.08 ± 0.02
RWW	0.86 ± 0.14	0.11 ± 0.02	0.56 ± 0.22	0.07 ± 0.03

**Figure 8:**
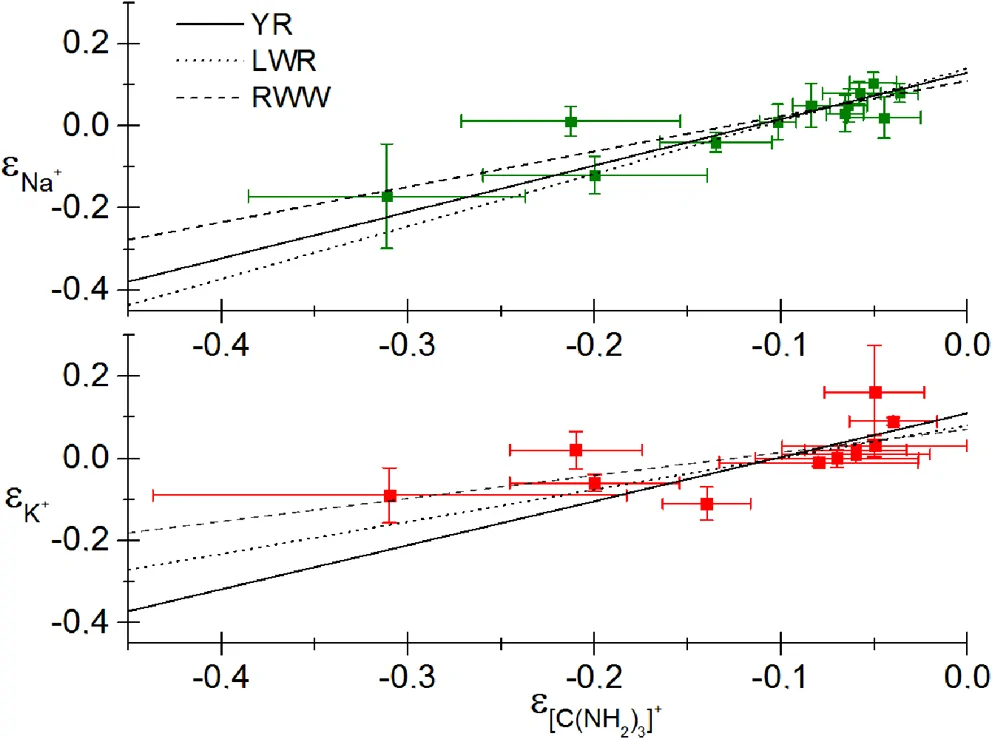
Plot of ε_K+,_ ε_Na+_, and ε_[C(NH2)3]+_(YR, York regression; LWR, linear wage regression; RWW, regression without wage).

Implementing these approaches leads to different a and b values (Table 6). However, only in the case of the York regression, the slopes obtained for both relationships, ε_Na^+^_ = f­(ε_[C(NH2)3]+_) and ε_K^+^_ = f­(ε_[C(NH2)3]+_), are found to be very similar. Consequently, the ε_[C(NH2)3]+_ values derived using this approach are significantly more consistent and, therefore, more reliable than those obtained using other methods. Due to the very limited number of available data for [N­(CH_3_)_4_]^+^ salts, analogous correlations could not be established. However, for bulky anions, such as Cl^–^, Br^–^, and I^–^, the ε values exhibit a linear dependence on the ionic radius of the cation M^+^ in MBr and MI electrolytes, as shown in Figure S11 for the Li^+^, Na^+^, K^+^, Rb^+^, Cs^+^, [C­(NH_2_)_3_]^+^ and [N­(CH_3_)_4_]^+^ salts. Given the linear dependence of the ε_M‑[Er(L)]_ (where M = Na, K and [C­(NH_2_)_3_]) parameter values on the cationic radius, the ε_[N(CH3)4]‑[Er(L)]_ parameter was extrapolated (see Table 5) and finally the ε_[N(CH3)4]‑[Er(L)(OH)]_ parameter was estimated using eq (9) and the data summarized in Table 5. Inorganic cations (Na^+^, K^+^) interact more strongly with the hydrolyzed [Er­(EDTA)­(OH)­(H_2_O)]^2–^ species than with the unhydrolyzed [Er­(EDTA)­(H_2_O)_2_]^−^ form. Furthermore, the interaction parameter ε_M‑[Er(L)]_ exhibits less scatter than ε_M‑[Er(L)(OH)]_, which varies over a considerably wider range. As shown by Simoes et al.,^96^ the more negative the Pitzer’s β^(0)^ parameter value, the stronger the association between cations and anions. According to the relation (12) derived by Grenthe et al.,^78^ such dependences should also be reflected in the *ε*_γ_ parameter values.

12 $$\beta^{\left(0\right)}-\frac{\varepsilon_{\gamma }}{2}\approx \mathrm{const}$$

The results may indicate that kosmotropic inorganic cations (Na^+^, K^+^) stabilize hydroxy complexes more effectively than bulky chaotropic organic cations ([C­(NH_2_)_3_]^+^ and [N­(CH_3_)_4_]^+^). Moreover, potassium cations, for which the enthalpy of hydration (−334 kJ·mol^–1^) is less negative than that of Na^+^ (−416 kJ·mol^–1^),^97^ may more readily release water molecules from their first coordination sphere and, as a result, can interact more directly with the complex anion than the other cations studied.

## Conclusions

The equilibrium hydrolysis reaction of the Er­(III)–EDTA system was systematically investigated in order to elucidate the influence of various ionic media (NaCl, KCl, [C­(NH_2_)_3_]­Cl, [N­(CH_3_)_4_]­Cl) on the stability, structure, and spectroscopic properties of the coordination complexes involved in the reaction.

Structural characterization of the following compounds was carried out: Na­[Er­(EDTA)­(H_2_O)_3_]·3.5H_2_O (**ErW3**), K­[Er­(EDTA)­(H_2_O)_2_]·4H_2_O (**ErW2**), K_5_[Er­(EDTA)­(μ-OH)]_2_Cl·5.5H_2_O (**Er2OH2**), [C­(NH_2_)_3_]_2_[Er­(EDTA)­(H_2_O)­F]·2H_2_O (**ErWF**), [C­(NH_2_)_3_]_3_[Er­(EDTA)­F_2_]·H_2_O (**ErF2**), and K_4_[Er­(EDTA)­(μ-F)]_2_·8H_2_O (**Er2F2**).

It was shown that the monomeric monofluoride (**ErWF**), monomeric difluoride (**ErF2**), and dimeric (**Er2F2**) eight-coordinate Er­(III) compounds can, to some extent, serve as mimetics of the Er­(III) hydroxy complex. However, differences in the interaction strengths between K^+^ and OH^–^ and F^–^ were observed, arising from the nonspherical geometry of the OH^–^ anion compared with the spherically symmetric F^–^ anion. Moreover, the OH^–^ anion exerts a stronger perturbing effect on the interaction between Er­(III) and the EDTA ligand because the strongly interacting OH^–^ anions pull the Er­(III) cation out of the EDTA cage. In the fluoride complexes under study, the Er­(III) ion is more tightly encapsulated within the resulting ternary complex, whereas the OH^–^ ligands weaken the Er–N and Er–O bonds compared with the F^–^ ligands. This may be attributed to the greater covalent character of the Er–OH bond relative to the Er–F bond.

Structural data of the monocrystals, combined with high-resolution UV–vis–NIR absorption spectroscopy of monocrystals and solutions, as well as ^89^Y NMR studies of solutions, provided insights into the identification of the M­(III) species present in the solution. The monohydroxide eight-coordinate [Er­(EDTA)­(OH)­(H_2_O)]^2–^ coordination complex was established as the predominant species in basic aqueous solutions.

The hydrolysis equilibrium constants were determined at varying ionic strengths and, together with supporting osmotic data, extrapolated to zero ionic strength using the Specific Ion Interaction Theory (SIT). The determined value of log β_OH_^o^ for the hydrolysis reaction was 1.73 ± 0.23.

Analysis of both log β_OH_ and ε values, as well as the observed changes in the spectroscopic features of the f–f transitions, reveals that Na^+^ and K^+^ cations interact more strongly with the hydrolyzed complex [Er­(EDTA)­(OH)­(H_2_O)]^2–^ than with the organic [C­(NH_2_)_3_]^+^ and [N­(CH_3_)_4_]^+^ cations. Among these cations, Na^+^ and K^+^ exhibit the strongest stabilizing effects on the hydrolyzed [Er­(EDTA)­(OH)­(H_2_O)]^2–^ species. A similar strong influence was observed for the CsCl solutions. The association constants of the {M^+^···[Er­(EDTA)­(OH)­(H_2_O)]^2–^} ionic pair were determined to be β_Na_ = 0.34 ± 0.09 (for NaCl), β_K_ = 0.6 ± 0.07 (for KCl), and β_Cs_ = 0.5 ± 0.1 (for CsCl). This is likely attributed to the favorable spatial fitting of these cations within the secondary coordination sphere of the [Er­(EDTA)­(OH)­(H_2_O)]^2–^ complex. Finally, this interaction effectively shifts the hydrolysis equilibrium toward hydroxy complex formation.

The results may indicate that kosmotropic inorganic cations (Na^+^, K^+^) stabilize hydroxy complexes more effectively than bulky chaotropic organic cations ([C­(NH_2_)_3_]^+^ and [N­(CH_3_)_4_]^+^). Moreover, potassium cations, for which the enthalpy of hydration is smaller than that of Na^+^, may more readily release water molecules from their nearest coordination sphere. As a result, this cation interacts more directly with the complex anion than the other cations under study. Finally, the scattered logβ values for hydrolysis reactions reported in the literature originate from differences in the specific interactions of the cations in the ionic medium, which, in turn, significantly perturb the stability of the hydroxo complexes.

## Supplementary Material



## Notes

Selected structural data of crystals under study (**Table S1**); hydrogen-bond geometries (Å, °) for Na­[Er­(EDTA)­(H_2_O)_3_]·3.5H_2_O, K­[Er­(EDTA)­(H_2_O)_2_]·4H_2_O, K_5_[Er_2_(EDTA)_2_(OH)_2_]­Cl·5.5H_2_O, [C­(NH_2_)_3_]_2_[Er­(EDTA)­(F)­(H_2_O)]·2H_2_O, [C­(NH_2_)_3_]_3_[Er­(EDTA)­(F)_2_]·H_2_O, K_4_[Er_2_(EDTA)_2_(F)_2_]·4H_2_O (**Tables S2a, S2b, S2c**, **S2d, S2e**, and **S 2f**); oscillator strengths (*P*·10^8^) and Ω_λ_ parameter values for the crystals (colorless columns) and solutions (**Table S3);** experimental data used for determining the osmotic coefficient and ε_γ_ parameter for the Na^+^–[Er­(EDTA)]^−^ and K^+^–[Er­(EDTA)]^−^ ionic pairs (**Tables S4a, S4b)**; theoretical and experimental X-ray powder diffractogram of the [C­(NH_2_)_3_]_2_SO_4_ crystal, the experimental recorded at RT, and theoretical generated for solved structure measured at 100 K (**Figure S1)**; EDS spectra of Er_2_OH_2_ (**Figure S2);** absorption spectra of the ^4^I_15/2_″^2^H_11/2_ and ^4^I_15/2_″^4^S_3/2_ transitions of the Er­(III)–EDTA system recorded during gravimetric titrations of the basic solutions of Er­(III)–EDTA–OH with solid NaCl, KCl, and CsCl (**Figure S3)**; correlations between the interaction coefficients in chloride, bromide, iodide, and [Er­(EDTA)]^−^ media for monovalent cations (**Figure S4)** (PDF)

## References

[1] Hehlen M. P., Güdel H. U. (1993). Optical
spectroscopy of the dimer system Cs_3_Yb_2_Br_9_. J. Chem. Phys..

[2] Schugar H. J., Solomon E. I., Cleveland W. L., Goodman L. (1975). Simultaneous Pair Electronic
Transitions in Yb_2_O_3_. J. Am. Chem. Soc..

[3] Janicki R., Eggen M., Kędziorski A., Ziobro M., Rok M., Korabik M., Krośnicki M. (2025). A structurally
elusive phase transition
with significant physicochemical consequences–the case of the
[C­(NH_2_)_3_]_3_[Dy­(EDTA)­(CO_3_)]·H_2_O crystal. J. Mater. Chem.
C.

[4] Boldyrev K. N., Malkin B. Z., Popova M. N. (2022). Observation of the hyperfine structure
and anticrossings of hyperfine levels in the luminescence spectra
of LiYF_4_:Ho^3+^. Light Sci.
Appl..

[5] Nielsen V. R. M., Sørensen T. J. (2025). Electronic
energy levels and optical
spectra of trivalent lanthanide ions in water – Ce­(III), Pr­(III),
Nd­(III), Sm­(III), Eu­(III), Gd­(III), Tb­(III), Dy­(III), Ho­(III), Er­(III),
Tm­(III), and Yb­(III). Nature Commun..

[6] Lindqvist-Reis P., Klenze R., Schubert G., Fanghänel T. (2005). Hydration
of Cm^3+^ in Aqueous Solution from 20 to 200 °C. A Time-Resolved
Laser Fluorescence Spectroscopy Study. J. Phys.
Chem. B.

[7] Janicki R., Mondry A., Starynowicz P. (2017). Carboxylates of rare earth elements. Coord. Chem. Rev..

[8] Mondry A., Starynowicz P. (1998). Crystal structure and absorption
spectroscopy of a
dimeric neodymium­(III) complex with triethylenetetraaminehexaacetic
acid (H_6_ttha), Na0.5H_5.5_[Nd_2_(ttha)_2_]·7.5NaClO_4_·16.83H_2_O. J. Chem. Soc., Dalton Trans..

[9] Mondry A. (1989). Spectroscopic
properties of Ho^3+^ complexes with dipicolinic acid in solution
and single crystals. Inorg. Chim. Acta.

[10] Janicki R., Gałęzowska J., Mondry A. (2017). Stoichiometry of lanthanide
(III) complexes with tripodal aminophosphonic ligands – a new
solution to an old problem. Inorg. Chem. Front..

[11] Janicki R., Starynowicz P., Mondry A. (2011). Lanthanide carbonates. Eur. J.
Inorg. Chem..

[12] Lindqvist-Reis P., Réal F., Janicki R., Vallet V. (2018). Unraveling the Ground
State and Excited State Structures and Dynamics of Hydrated Ce^3+^ Ions by Experiment and Theory. Inorg.
Chem..

[13] Janicki R., Modry A. (2019). Structural and thermodynamic aspects of hydration of Gd­(III) systems. Dalton Trans..

[14] Janicki R., Siczek M., Starynowicz P. (2024). In Search
of Covalency Measure of
Gd­(III)-Ligand Interactions. J. Phys. Chem.
Lett..

[15] Gałęzowska J., Janicki R., Kozłowski H., Mondry A., Młynarz P., Szyrwiel L. (2010). Unusual Coordination Behaviour of a Phosphonate-and
Pyridine-Containing Ligand in a Stable Lanthanide Complex. Eur. J. Inorg. Chem..

[16] Janicki R., Mondry A. (2015). Thermodynamics of the hydration equilibrium derived
from the luminescence spectra of the solid state for the case of the
Eu–EDTA system. Phys. Chem. Chem. Phys..

[17] Mondry A., Starynowicz P. (2006). Ten-Coordinate
Neodymium­(III) Complexes with Triethylenetetraaminehexaacetic
Acid. Eur. J. Inorg. Chem..

[18] Janicki R., Lindqvist-Reis P. (2018). Eu­(III) and
Cm­(III) tetracarbonates–in the quest
for the limiting species in solution. Dalton
Trans..

[19] Janicki R., Starynowicz P., Mondry A. (2008). Complexes of Yb^3+^ with
EDTA and CDTA – Molecular and Electronic Structure. Eur. J. Inorg. Chem..

[20] Okemgbo A. A., Hill H. H., Metcalf S. G., Hachelor M. (1999). Metal ion interferences
in reverse polarity capillary zone electrophoretic analysis of Hanford
Defense Waste for ethylenediaminetetraacetic acid (EDTA) and n-hydroxyethylethylenediaminetriacetic
acid (HEDTA). Anal. Chim. Acta.

[21] Thakur P., Conca J. L., Choppin G. R. (2012). Mixed Ligand
Complexes of Am^3+^, Cm^3+^ and Eu^3+^ with
HEDTA and HEDTA
+ NTA-Complexation Thermodynamics and Structural Aspects. J. Solution Chem..

[22] DiBlasi N. A., Tasi A. G., Trumm M., Schnurr A., Gaona X., Fellhauer D., Dardenne K., Rothe J., Reed D. T., Hixon A. E., Altmaier M. (2022). Pu­(III) and Cm­(III)
in the presence
of EDTA: aqueous speciation, redox behavior, and the impact of Ca­(II). RSC Adv..

[23] Merbach A., Gnaegi F. (1969). Nuclear magnetic resonace study of scandium, yttrium,
lanthanum and lutetium ethylenediaminetetraacetates. Chimia.

[24] Southwood-Jones R. V., Merbach A. E. (1978). Nuclear Magnetic Resonance Study of Preseodymium, Europium
and Ytterbium Ethylenediaminetetraacetates. Inorg. Chim. Acta.

[25] Martynenko L. I., Spitsyn V. I., Artyukhi G. A. (1970). Influence of citric
acid on ion exchange
of rare earth elements between cationite and solution of ethylenediaminetetraacetic
acid. Z. Neorgan. Khim..

[26] Eckardt D., Holleck L. (1955). Zur Komplexchemie der
2- und 3 wertigen Seltenen Erden
Polarographische Untersuchungen an Europiumkomplexen. Z. Elektrochem..

[27] Felmy, A. R. ; Wang, Z. ; Dixon, D. A. ; Joly, A. G. ; Rustad, J. R. ; Mason, M. J. The aqueous complexation of Eu­(III) with organic chelates at high-base concentration: Molecular and thermodynamic modeling results. In Nuclear Site Remediation; ACS, 2001; Vol. 778, pp 63–82.

[28] Krivonogikh T. S., Pyreu D. F., Kozlovskii E. V. (2010). Thermodynamics
of mixed-ligand complexation
of gadolinium ethylenediaminetetraacetate with amino acids in aqueous
solution. Koord. Khim..

[29] Janicki R., Mondry A. (2019). Structural and thermodynamic aspects
of water–carbonate
exchange equilibrium for MIII/IV–EDTA–carbonate systems. Inorg. Chem. Front..

[30] Hummel, W. Organic Complexation of Radionuclides in Cement Pore Water: A Case Study. Technical Report TM-41–93–03; Paul Scherrer Institut: Villigen, Switzerland; 1993; pp 65-75.

[31] Shalinets A. B. (1972). Investigation
of the Complex Formation of Trivalent Actinide and Lanthanide Elements
by the Method of Electromigration XVI Ethylenediaminetetraacetic Acid. Radiokhimiya.

[32] Brønsted J. N. (1922). Studies
on solubility. IV. The principle of the specific interaction of ions. J. Am. Chem. Soc..

[33] Guggenheim E. A. (1935). The specific
thermodynamic properties of aqueous solutions of strong electrolytes. Philos. Mag..

[34] Scatchard G. (1936). Concentrated
solutions of strong Electrolytes. Chem. Rev..

[35] Grenthe, I. ; Mompean, F. ; Spahiu, K. ; Wanner, H. , Guidelines for the extrapolation to zero ionic strength OECD Nuclear Energy Agency, Data Bank, 2013.

[36] Grenthe I., Plyasunova A. (1997). On the use
of semiempirical electrolyte theories for
the modeling of solution chemical data. Pure
Appl. Chem..

[37] Sheldrick G. M. (2015). SHELXT
– Integrated space-group and crystal-structure determination. Acta Crystallogr., Sect. A.

[38] Sheldrick G. M. (2015). Crystal
structure refinement with. SHELXL, Acta Cryst.
Sect. C.

[39] Dolomanov O. V., Bourhis L. J., Gildea R. J., Howard J. A. K., Puschmann H. (2009). OLEX2: a complete
structure solution, refinement and analysis program. J. Appl. Crystallogr..

[40] Holmberg R. J., Korobkov I., Murugesu M. (2016). Enchaining EDTA-chelated
lanthanide
molecular magnets into ordered 1D networks. RSC Adv..

[41] Eggen M., Kędziorski A., Janicki R., Korabik M., Krosnicki M. (2022). The ab initio
and experimental study of the spectroscopic and magnetic properties
of Ho­(III)-EDTA. Polyhedron.

[42] Zhuravlev M. G., Sergeev A. V., Mistriukov V. E., Mikhailov Yu. N., Shchelokov R. N. (1989). Mixed ethylenediaminetetraacetatofluorides
of lanthanides
(3) and actinides (4). roc. Nat. Acad. Sci.
USSR.

[43] Andrews, L. C. ; McMullen, R. K. CSD Communication. (Private Communication)2019; Vol. 406 10.1016/S0140-6736(25)01637-X.

[44] Judd B. R. (1962). Optical
absorption intensities of rare-earth ions. Phys.
Rev..

[45] Ofelt G. S. (1962). Intensities
of crystal spectra of rare-earth. J. Chem. Phys..

[46] Carnall W. T., Fields P. R., Wybourne B. G. (1965). Spectral
Intensities of the Trivalent
Lanthanides and Actinides in Solution. I. Pr^3+^, Nd^3+^, Er^3+^, Tm^3+^, and Yb^3+^. J. Chem. Phys..

[47] Modrzejewski, B. , Pomiary pH, WNT, Warszawa, 1971.

[48] Buck R. P., Rondinini S., Covington A. K., Baucke F. G. K., Brett C. M. A., Camoes M. F., Milton M. J. T., Mussini T., Naumann R., Pratt K. W., Spitzer P., Wilson G. S. (2002). Measurement of pH.
Definition, standards, and procedures (IUPAC Recommendations 2002). Pure Appl. Chem..

[49] Rossberg A., Reich T., Bernhard G. (2003). Complexation
of uranium­(VI) with
protocatechuic acid-application of iterative transformation factor
analysis to EXAFS spectroscopy. Anal. Bioanal.
Chem..

[50] Zhu Y., Luo F., Song Y., Feng X., Luo M., Liao Z., Sun G., Tian X., Yuan Z. J. (2012). The First One-Pot Synthesis of Multinuclear
3d-4f Metal–Organic Compounds Involving a Polytopic N,O-Donor
Ligand Formed in Situ. Cryst. Grow. Des..

[51] Kratsch J., Beele B. B., Koke C., Denecke M. A., Geist A., Panak P. J., Roesky P. W. (2014). 6-(Tetrazol-5-yl)-2,
2′-bipyridine:
A highly selective ligand for the separation of lanthanides­(III) and
actinides­(III). Inorg. Chem..

[52] Walker, J. B. ; Choppin, G. R. Thermodynamic Parameters of Fluoride Complexes of the Lanthanides 1967; pp 127–140.

[53] Ramírez-García J. J., Solache-Rios M., Jimenez-Reyes M., Rojas-Hernandez A. (2003). Solubility
and hydrolysis of La, Pr, Eu, Er, and Lu in 1 M NaCl ionic strength
at 303 K. J. Sol. Chem..

[54] Parker M. L., Jian J., Gibson J. K. (2021). Bond dissociation
energies of low-valent
lanthanide hydroxides: lower limits from ion–molecule reactions
and comparisons with fluorides. Phys. Chem.
Chem. Phys..

[55] Ślepokura K., Cabreros T. A., Muller G., Lisowski J. (2021). Sorting Phenomena and
Chirality Transfer in Fluoride-Bridged Macrocyclic Rare Earth Complexes. Inorg. Chem..

[56] Starynowicz P., Lisowski J. (2019). Chirality transfer
between hexaazamacrocycles in heterodinuclear
rare earth complexes. Dalton Trans..

[57] Janicki R., Mondry A. (2014). A new approach to determination
of hydration equilibria
constants for the case of [Er­(EDTA)­(H_2_O)_n_]^−^ complexes. Phys. Chem. Chem.
Phys..

[58] Király R., Tóth I., Brücher E. (1981). Aminopolycarboxylates of rare earths-VI:
Determination of stability constants and formation enthalpies of rare
earth­(III)-Ethylenediamine tetraacetate-Fluoride mixed ligand complexes. J. Inorg. Nucl. Chem..

[59] Janicki R., Siczek M., Starynowicz P. (2024). In Search
of Covalency Measure of
Gd­(III)-Ligand Interactions. J. Phys. Chem.
Lett..

[60] Janicki R., Mondry A. (2013). Relationships between structure and spectroscopic properties
of Nd^3+^ ethylenediaminetetramethylenephosphonates and ethylenediaminetetraacetates. Eur. J. Inorg. Chem..

[61] Peacock R. D. (1975). The Intensities
of Lanthanide f–f Transitions. Struct.
Bonding (Berlin).

[62] Mason S. F. (1984). Independent-systems
mechanisms for f–f transition probabilities in lanthanide coordination
compounds. Inorg. Chim. Acta.

[63] Poon Y. M. (1984). and D.
J. Newman, Overlap and covalency contributions to lanthanide-ion spectral
intensity parameters. J. Phys. C: Solid State
Phys..

[64] Jørgensen C. K., Judd B. R. (1964). Hypersensitive pseudoquadrupole transitions in lanthanides. Mol. Phys..

[65] Görller-Walrand, C. ; Binnemans, K. Handbook on the Physics and Chemistry of Rare Earths; Gschneidner, K. A., Jr. ; Eyring, L. , Eds.; Elsevier, 1998; Vol. 25, pp 101–264.

[66] Smentek L., Kędziorski A. (2009). f↔ f electric dipole transitions; old problems
in a new light. J. Alloys Compd..

[67] Xing Y., Jindal A. K., Regueiro-Figueroa M., Le Fur M., Kervarec N., Zhao P., Kovacs Z., Valencia L., Pørez-Lourido P., Tripier R., Esteban-Gómez D., Platas-Iglesias C., Sherry A. D. (2016). The Relationship between NMR Chemical
Shifts of Thermally
Polarized and Hyperpolarized ^89^Y Complexes and Their Solution
Structures. Chem.—Eur. J..

[68] Bodman S. E., Butler S. J. (2021). Advances in anion
binding and sensing using luminescent
lanthanide complexes. Chem. Sci..

[69] Ekberg, Ch. ; Brown, P. L. , Hydrolysis of Metal Ions; Willey 2016, pp 61–130.

[70] Macaskill J. B., Robinson R. A., Bates R. G. (1977). Osmotic
Coefficients and Activity
Coefficients of Guanidinium Chloride in Concentrated Aqueous Solutions
at 25°C. J. Chem. Eng. Data.

[71] El
Guendouzi M., Marouani M. (2003). Water activities and osmotic and
activity coefficients of aqueous solutions of nitrates at 25°C
by the hygrometric method. J. Sol. Chem..

[72] Marcus Y. (1993). Thermodynamics
of solvation of ions. Part 6.-The standard partial molar volumes of
aqueous ions at 298.15 K. J. Chem. Soc. Farad.
Trans..

[73] Marcus Y. (2012). The guanidinium
ion. J. Chem. Therm..

[74] Das B. (2004). Pitzer ion
interaction parameters of single aqueous electrolytes at 25°C. J. Sol. Chem..

[75] Ciavatta L. (1980). The Specific
Interaction Theory in the Evaluating Ionic Equilibria. Ann. Chim. (Rome).

[76] Pitzer, K. S. , Activity coefficients in electrolyte solutions, CRC Press, Boca Raton, Fla, 1991; pp 75–147.

[77] Hummel, W. ; Anderegg, G. ; Rao, L. ; Puigdomènech, I. ; Tochiyama, O. Chemical Thermodynamics of Compounds and Complexes of U, Np, Pu, Am, Tc, Se, Ni and Zr with Selected Organic Ligands. In Chemical Thermodynamics Series; OECD Nuclear Energy Agency: Paris, France, 2005; Vol. 9.

[78] Grenthe, I. ; Puigdomenech, I. Modelling in Aquatic Chemistry, 1997; pp 325–493.

[79] Ciavatta L. (1980). The specific
interaction theory in evaluating ionic equilibria. Ann. Chim..

[80] Sarbar M., Covington A. K., Nuttall R. L., Goldberg R. N. (1982). The activity and
osmotic coefficients of aqueous sodium bicarbonate solutions. J.Chem. Therm..

[81] Roy R. N., Gibbons J. J., Williams R., Godwin L., Baker G. (1984). The thermodynamics of aqueous carbonate solutions II.
Mixtures of
potassium carbonate, bicarbonate, and chloride. J. Chem. Therm..

[82] Abdel-Salam O. E. (1982). Activity
Coefficients of Tetramethylammonium Carbonate, Bicarbonate and Hydroxide
in Aqueous Medium. Indian J. Chem..

[83] Miyajima K., Inamura K., Hamaguchi T., Yoshida H., Nakagaki M. (1975). Activity coefficients
and partial molar volumes of guanidinium salts in aqueous solution
at 25°C 1. Bull. Chem. Soc. Jpn..

[84] Bonner O. D. (1977). Hydrogen
bonding in guanidinium fluoride. J. Phys. Chem.
A.

[85] Pitzer, K. S. ; Press, C. R. C. Activity Coefficients In Electrolyte Solutions; CRC press: Boca Raton, FL, 1991; Vol. 2, p 102.

[86] Bonner O. D. (1976). J. Chem. Therm..

[87] Robinson R. A., Macaskill J. B. (1979). Osmotic
coefficients of aqueous sodium carbonate solutions
at 25°C. J. Sol. Chem..

[88] Sarbar M., Covington A. K., Nuttall R. L., Goldberg R. N. (1982). Activity and osmotic
coefficients of aqueous potassium carbonate. J. Chem. Therm..

[89] Kumar A. (2001). Aqueous guanidinium
salts: Part II. Isopiestic osmotic coefficients of guanidinium sulphate
and viscosity and surface tension of guanidinium chloride, bromide,
acetate, perchlorate and sulphate solutions at 298.15°K. Fluid Phase Equilib..

[90] Robinson, R. A. ; Stokes, R. H. Electrolyte Solutions, London, 1959; pp 483–490.

[91] Tsurko E. N., Lange T., Betz K., Stauber J., Schwarz S., Kunz W., Müller R. (2026). Thermodynamic
Description of Aqueous
Mixtures of New Antifreezes. J. Chem. Eng. Data.

[92] Miyajima K., Yoshida H., Nakagaki M. (1978). Studies on
the Aqueous Solutions
of Guanidinium Salts. VII. Activity Coefficients and Partial Molar
Volumes for Guanidinium Salts with Hydrophobic Counter Anions in Aqueous
Solutions. Bull. Chem. Soc. Jpn..

[93] Desnoyers J. E., Pagé R., Perron G., Fortier J.-L., Leduc P.-A., Platford R. F. (1973). Thermodynamic
and transport properties of sodium benzoate
and hydroxy benzoates in water at 25°C. Can. J. Chem..

[94] York D., Evensen N. M., Martínez M. L., De Basabe
Delgado J. (2004). Unified equations for the slope, intercept, and standard
errors of the best straight line. Am. J. Phys..

[95] Cantrell C. A. (2008). Review
of methods for linear least-squares fitting of data and application
to atmospheric chemistry problems. Atmos. Chem.
Phys..

[96] Simoes M. C., Hughes K. J., Ingham D. B., Ma L., Pourkashanian M. (2016). Estimation
of the Pitzer Parameters for 1–1, 2–1, 3–1, 4–1,
and 2–2 Single Electrolytes at 25°C. J. Chem. Eng. Data.

[97] Marcus, Y. Ions in Solution and their Solvation; John Wiley & Sons, 2015; p 113.

