# Supplementary material for: Role of Ionic Media in f–f Transitions, Stability, and the Structure of Ln(III) Coordination Compounds
Source: Inorg Chem. 2026 Aug 10;65(33):19221–35. doi: 10.1021/acs.inorgchem.6c02135 (PMC13509000; doi:10.1021/acs.inorgchem.6c02135)
Supplement: Supplementary file 1 [file ic6c02135_si_001.pdf]

SUPPORTING INFORMATION

Role of Ionic Media in f–f Transitions, Stability and the Structure of Ln(III) Coordination Compounds

Marcin Ziobro, Rafał Janicki*

AUTHOR INFORMATION

[a] University of Wrocław, Faculty of Chemistry, F. Joliot Curie 14, 50-383 Wrocław.

**Table S1.**		Selected structural data of crystals under study…………………………	3
**Table S2.**		Selected bond length (Å) determined for ErW3, ErW2, Er2OH2, ErWF, ErF2, Er2F2…………………………………………………………	4
**Table S3a**		Hydrogen-bond geometry (Å, °) for $Na[Er(EDTA)(H_2O)_3]\cdot 3.5H_2O$….....	5
**Table S3b.**		Hydrogen-bond geometry (Å, °) for $K[Er(EDTA)(H_2O)_2]\cdot 4H_2O$………..	6
**Table S3c.**		Hydrogen-bond geometry (Å, °) for $K_5[Er_2(EDTA)_2(OH)_2]Cl\cdot 5.5H_2O$....	6
**Table S3d.**		Hydrogen-bond geometry (Å, °) for $[C(NH_2)_3]_2[Er(EDTA)(F)(H_2O)]\cdot 2H_2O$…………………………………..	7
**Table S3e.**		Hydrogen-bond geometry (Å, °) for $[C(NH_2)_3]_3[Er(EDTA)(F)_2]\cdot H_2O$…..	8
**Table S3f.**		Hydrogen-bond geometry (Å, °) for $K_4[Er_2(EDTA)_2(F)_2]\cdot 4H_2O$………...	9
**Table S4.**		Oscillator strengths ($P\cdot 10^8$) and Ω_λ parameter values for the crystals (colourless columns) and solutions (grey columns) (*P* values of hypersensitive transitions are in bold)……………………………………	10
**Table S5.**		$(\sum \frac{\alpha}{r^4})^2$ factor for coordinated coligands in the Er(III)-EDTA complex and the corresponding Judd–Ofelt Ω_2 parameter values	11
**Table S6a.**		Experimental data used for determination of osmotic coefficient and ε_γ parameter for Na^+ - $[Er(EDTA)]^-$ ionic pair…………………………...	12
**Table S6b.**		Experimental data used for determination of osmotic coefficient and ε_γ parameter for K^+ - $[Er(EDTA)]^-$ ionic pair………………………………	12
**Figure S1.**		Experimental (•) and calculated (—) mole fractions of $[Er(EDTA)(H_2O)_2]^-$ species as a function of pH in NaCl electrolyte…….	13
**Figure S2.**		Experimental (•) and calculated (—) mole fractions of $[Er(EDTA)(H_2O)_2]^-$ species as a function of pH in NaCl electrolyte…….	14
**Figure S3.**		Experimental (•) and calculated (—) mole fractions of $[Er(EDTA)(H_2O)_2]^-$ species as a function of pH in KCl electrolyte……...	15
**Figure S4.**		Experimental (•) and calculated (—) mole fractions of $[Er(EDTA)(H_2O)_2]^-$ species as a function of pH in $[C(NH_2)_3]Cl$ electrolyte…………………………………………………………...	16
**Figure S5.**		Experimental (•) and calculated (—) mole fractions of $[Er(EDTA)(H_2O)_2]^-$ species as a function of pH in $[N(CH_3)_4]Cl$ electrolyte…………………………………………………………	17
**Figure S6.**		^{89}Y NMR spectra of the Y(III)-EDTA complex at pH 7 and 12 and different ionic strengths………………………………………………..	18

**Figure S7.**		**S7.** Theoretical and experimental X-ray powder diffractogram of the $[C(NH_2)_3]_2SO_4$ crystal. Experimental recorded at RT, theoretical generated for solved structure measured at 100 K………………………	19
**Figure S8.**		EDS spectra of the Er2OH2……………………………………………...	20
**Figure S9.**		Absorption spectra of the $4I_{15/2} \rightarrow {}^2H_{11/2}$ and $4I_{15/2} \rightarrow {}^4S_{3/2}$ recorded for $K[Er(EDTA)(H_2O)_2]\cdot 4H_2O$ and $[C(NH_2)_3]_2[Er(EDTA)(H_2O)_2]ClO_4\cdot 6H_2O$ crystals and $[Er(EDTA)(H_2O)_2]^-$ solution at pH = 7…………………………………...	21
**Figure S10.**		Absorption spectra (initial – red, final – blue) of the ${}^4I_{15/2}\rightarrow{}^2H_{11/2}$ and ${}^4I_{15/2}\rightarrow{}^4S_{3/2}$ transitions of the Er(III)-EDTA system recorded during gravimetric titrations of the basic solutions of Er(III)-EDTA-OH with solid NaCl, KCl and CsCl………………………………………………..	23
**Figure S11.**		The correlations between interaction coefficients in chloride, bromide, iodide and $[Er(EDTA)]^-$ media for monovalent cations………………….	23
**Figure S12.**		Plot of osmotic coefficient vs. ionic strengths for Na-Er(EDTA) and K-Er(EDTA) solutions……………………………………………………	24

Table S1. Selected structural data of crystals under study

	$Na[Er(EDTA)(H_2O)_3]\cdot 3.5H_2O$	$K[Er(EDTA)(H_2O)_2]\cdot 4H_2O$	$[C(NH_2)_3]_2[Er(EDTA)(H_2O)F]\cdot 2H_2O$	$[C(NH_2)_3]_3[Er(EDTA)F_2]\cdot H_2O$	$K_4[Er(EDTA)(\mu\text{-}F)]_2\cdot 8H_2O$	$K_5[(Er(EDTA)(\mu\text{-}OH)]_2Cl\cdot 5.5H_2O$	$[C(NH_2)_3]_2SO_4$
Chemical formula	$C_{10}H_{25}ErN_2NaO_{14.50}$	$C_{10}H_{24}ErKN_2O_{14}$	$C_{12}H_{30}ErFN_8O_{11}$	$C_{13}H_{32}ErF_2N_{11}O_9$	$C_{10}H_{20}ErFK_2N_2O_{12}$	$C_{20}H_{35}ClEr_2K_5N_4O_{23.50}$	$C_2H_{12}N_6O_4S$
M_r	595.57	602.67	648.70	691.75	624.74	1272.00	216.24
Crystal system, space group	Orthorhombic, *Fdd*2	Monoclinic, *P*2/*n*	Triclinic, *P*	Monoclinic, $P2_1/n$	Triclinic, *P*	Monoclinic, I2/m	Cubic, $P4_332$
Temperature (K)	100	100	100	100	100	100	100
a, *b*, *c* (Å)	34.665 (6), 18.746 (4), 12.040 (2)	8.659 (2), 25.821 (3), 8.722 (2)	9.517 (6), 11.828 (8), 12.237 (8)	13.411 (3), 10.408 (2), 17.509 (3)	8.804 (2), 9.330 (2), 12.765 (3)	8.785 (2), 24.223 (6), 9.050 (3)	17.797 (4)
α, β, γ (°)	90, 90, 90	90, 97.54 (2), 90	64.08(7), 70.05(6), 68.09 (6)	90, 101.19 (2), 90	87.23 (2), 82.81 (2), 65.42 (2)	90, 97.06 (3), 90	90
V (Å^3)	7824.1 (3)	1933.2 (7)	1122.9 (15)	2397.5 (8)	946.0 (4)	1911.2 (9)	5637 (4)
Z	16	4	2	4	2	2	20
Radiation type	Mo *K*α	Mo *K*α	Mo *K*α	Mo *K*α	Mo *K*α	Mo Kα	Cu *K*α
μ (mm^{-1})	4.39	4.63	3.82	3.58	4.95	5.07	2.63
Crystal size (mm)	0.20 × 0.13 × 0.10	0.33 × 0.19 × 0.09	0.47 × 0.34 × 0.06	0.41 × 0.12 × 0.08	0.08 × 0.06 × 0.02	0.32 × 0.04 × 0.03	0.32 × 0.24 × 0.10
Diffractometer	XtaLAB Synergy R, DW system, HyPix-Arc 150	Xcalibur, Ruby, Gemini ultra	Xcalibur, Ruby, Gemini ultra	Xcalibur, Ruby, Gemini ultra	XtaLAB Synergy R, DW system, HyPix-Arc 150	Xcalibur, Ruby, Gemini ultra	XtaLAB Synergy R, DW system, HyPix-Arc 150
Absorption correction	Gaussian	Analytical	Analytical	Gaussian	Gaussian	Analytical	0.588, 0.839
T_{min}, T_{max}	0.581, 1.000	0.409, 0.681	0.367, 0.846	0.490, 0.771	0.777, 0.988	0.510, 0.872	0.581, 1.000
No. of measured, independent and observed [$I > 2\sigma(I)$] reflections	20884, 5701, 5377	18813, 5452, 5018	9182, 5132, 4948	11251, 5472, 4782	16140, 5685, 5208	4140, 2235, 2156	106553, 1905, 1874
R_{int}	0.053	0.018	0.021	0.032	0.050	0.023	0.027
$(\sin \theta/\lambda)_{max}$ (Å^{-1})	0.714	0.712	0.679	0.679	0.714	0.678	0.621
$R[F^2 > 2\sigma(F^2)]$, $wR(F^2)$, S	0.027, 0.059, 1.03	0.021, 0.041, 1.19	0.019, 0.044, 1.07	0.029, 0.066, 1.06	0.028, 0.063, 1.01	0.019, 0.044, 1.08	0.020, 0.057, 1.15
No. of reflections	5701	5452	5132	5472	5685	2235	1905
No. of parameters	315	305	318	334	285	175	104
H-atom treatment	H atoms treated by a mixture of independent and constrained refinement	H atoms treated by a mixture of independent and constrained refinement	H atoms treated by a mixture of independent and constrained refinement	H atoms treated by a mixture of independent and constrained refinement	H atoms treated by a mixture of independent and constrained refinement	H atoms treated by a mixture of independent and constrained refinement	H atoms treated by a mixture of independent and constrained refinement
$\Delta\rho_{max}$, $\Delta\rho_{min}$ (e Å^{-3})	1.33, −1.38	0.64, −0.91	0.89, −0.66	1.47, −1.01	1.22, −0.84	1.03, −0.90	0.14, −0.23

Table S2. Selected bond length (Å) determined for **ErW3, ErW2, Er2OH2, ErWF, ErF2, Er2F2**

Bond	Bond length (Å)					
	**ErW3** $[Er(EDTA)(H_2O)_3]^-$	**ErW2*** $[Er(EDTA)(H_2O)_2]^-$	**Er2OH2** $[Er(EDTA)(OH)]_2^{2-}$	**ErWF** $[Er(EDTA)F(H_2O)]^{2-}$	**ErF2** $[Er(EDTA)F_2]^{3-}$	**Er2F2** $[Er(EDTA)F]_2^{2-}$
Er-N1	2.587(5)	2.557(2)<br>2.570(2)	2.673(2)	2.609(2)	2.634(3)	2.605(3)
Er-N2	2.672(6)	2.557(2)<br>2.570(2)	2.673(2)**	2.620(2)	2.589(3)	2.592(3)
Er-O1	2.364(4)	2.313(2)<br>2.290(2)	2.331(2)	2.297(2)	2.322(2)	2.302(2)
Er-O2	2.377(4)	2.280(2)<br>2.294(2)	2.311(2)	2.319(2)	2.327(2)	2.271(2)
Er-O3	2.369(4)	2.313(2)<br>2.290(2)	2.311(2)***	2.322(2)	2.334(2)	2.303(2)
Er-O4	2.345(4)	2.280(2)<br>2.294(2)	2.331(2)^{IV}*	2.298(2)	2.318(2)	2.282(2)
Er - F1/OH1/OW1	2.466(3)	2.346(2)<br>2.336(2)	2.279(2)	2.167(2)	2.208(2)	2.288(2)
Er - F2/OH2/OW2	2.416(4)	2.346(2)<br>2.336(2)	2.279(2)^{V}*	2.352(2)	2.176(2)	2.176(2)
Er-OW3	2.340(4)					

* in ErW2 crystal structure, two symmetrically independent Er sites are present
** symmetry-equivalent to the Er-N1 bond
*** symmetry-equivalent to the Er-O2 bond
^{IV}* symmetry-equivalent to the Er-O1 bond
^{V}* symmetry-equivalent to the Er-OH bond

Table S3a. Hydrogen-bond geometry (Å, °) for $Na[Er(EDTA)(H_2O)_3]\cdot 3.5H_2O$

D—H···*A*	*D*—H	H···*A*	*D*···*A*	*D*—H···*A*
Ow7A^A—Hw7A^A···Ow6	0.85(9)	2.08(10)	2.848(9)	150(7)
Ow1—Hw1A···Ow5	0.85(4)	1.95(5)	2.768(5)	164(6)
Ow1—Hw1B···Ow2	0.85(4)	2.66(5)	3.116(5)	115(4)
Ow1 —Hw1B···O3	0.85(4)	1.85(4)	2.685(5)	171(5)
Ow7A^A—Hw7B^A···O1	0.83(9)	2.35(10)	3.123(8)	157(8)
Ow7A^A—Hw7B^A···O2	0.83(9)	2.59(9)	3.058(8)	117(8)
Ow2—Hw2A···O4	0.85(3)	1.89(3)	2.726(5)	168(6)
Ow2—Hw2B···Ow5	0.84(4)	1.86(4)	2.686(5)	166(5)
Ow3—Hw3A···O7	0.84(5)	1.80(5)	2.629(6)	168(5)
Ow3—Hw3B···O8	0.85(5)	1.85(4)	2.656(5)	158(6)
Ow4—Hw4A···O5	0.84(11)	2.13(17)	2.834(5)	142(14)
Ow4—Hw4B···O5	0.85(12)	2.08(15)	2.834(5)	147(15)
Ow5—Hw5A···O6	0.84(5)	1.94(5)	2.727(5)	157(6)
Ow5—Hw5B···Ow4	0.83(4)	2.02(5)	2.843(7)	170(6)
Ow6—Hw6A···O8	0.82(6)	2.24(6)	3.022(6)	161(6)
Ow6—Hw6B···O6	0.82(6)	2.08(6)	2.798(6)	145(7)
C2—H2A···O5	0.99	2.58	3.506(8)	156
C2—H2B···O7	0.99	2.7	3.286(7)	118
C3—H3A···O6	0.99	2.42	3.397(7)	168
C3—H3B···O8	0.99	2.69	3.615(7)	155
C5—H5A···O8	0.99	2.61	3.564(7)	163
C9—H9A···Ow7B^B	0.99	2.69	3.528(18)	142

Table S3b. Hydrogen-bond geometry (Å, °) for $K[Er(EDTA)(H_2O)_2]\cdot 4H_2O$

D—H···*A*	*D*—H	H···*A*	*D*···*A*	*D*—H···*A*
Ow1—Hw1A···Ow3	0.80(4)	1.97(4)	2.746(3)	164(4)
Ow1—Hw1B···Ow4	0.70(4)	2.03(3)	2.721(3)	168(4)
Ow2—Hw2A···O11	0.80(4)	1.96(4)	2.726(2)	160(3)
Ow2—Hw2B···Ow5	0.78(4)	1.95(3)	2.719(3)	172(4)
Ow3—Hw3···O22	0.74(3)	2.09(3)	2.830(2)	173(4)
Ow4—Hw4A···O21	0.75(3)	2.00(3)	2.739(2)	169(3)
Ow4—Hw4B···O15	0.88(3)	1.94(3)	2.752(2)	151(3)
Ow5—Hw5A···O26	0.68(4)	2.23(4)	2.783(3)	139(4)
Ow5—Hw5B···Ow6	0.83(4)	2.03(4)	2.851(3)	168(4)
Ow6—Hw6···O12	0.77(4)	2.15(4)	2.919(2)	177(5)
Ow7—Hw7···O25	0.90(4)	1.98(4)	2.861(2)	168(4)
Ow8—Hw8···O16	0.85(4)	2.04(4)	2.870(2)	166(4)
C11—H11A···O16	0.99	2.43	3.391(3)	162
C13—H13B···Ow8	0.99	2.7	3.603(3)	152
C15—H15A···O15	0.99	2.38	3.289(3)	153
C22—H22A···O26	0.99	2.44	3.360(3)	154
C23—H23B···Ow7	0.99	2.72	3.622(3)	152
C25—H25A···O25	0.99	2.44	3.403(3)	163

Table S3c. Hydrogen-bond geometry (Å, °) for $K_5[Er_2(EDTA)_2(OH)_2]Cl\cdot 5.5H_2O$

D—H···*A*	*D*—H	H···*A*	*D*···*A*	*D*—H···*A*
Ow2—Hw2···O6	0.84(4)	2.03(4)	2.860(3)	170(5)
Ow3—Hw3···O2	0.84(3)	2.06(3)	2.856(2)	158(3)
C1—H1A···O6	0.99	2.49	3.429(3)	157
C5—H5A···O5	0.99	2.61	3.496(4)	150
Ow2—Hw2···O6	0.84(4)	2.03(4)	2.860(3)	170(5)
Ow3—Hw3···O2	0.84(3)	2.06(3)	2.856(2)	158(3)
C1—H1A···O6	0.99	2.49	3.429(3)	157
C5—H5A···O5	0.99	2.61	3.496(4)	150

Table S3d. Hydrogen-bond geometry (Å, °) for $[C(NH_2)_3]_2[Er(EDTA)(F)(H_2O)]\cdot 2H_2O$

D—H···*A*	*D*—H	H···*A*	*D*···*A*	*D*—H···*A*
Ow—Hw1···O3	0.79(5)	1.99(4)	2.775(4)	169(4)
Ow—Hw2···O6	0.77(4)	1.92(4)	2.689(4)	174(3)
Ow2—Hw21···O7	0.85	2.18	2.961(4)	152
N21—H21A···F	0.88	1.97	2.764(4)	149
N21—H21B···O2	0.88	2.03	2.885(3)	164
Ow2—Hw22···O7	0.86	1.89	2.726(3)	166
N22—H22A···N31	0.88	2.51	3.295(4)	149
N22—H22B···O6	0.88	2.11	2.947(4)	159
N23—23A···O5	0.88	2.05	2.857(4)	152
N23—H23B···Ow3	0.88	2.00	2.869(4)	169
Ow3—Hw31···F	0.85(4)	1.83(4)	2.676(3)	172(4)
N31—H31A···O5	0.88	1.95	2.828(4)	173
N31—H31B···O4	0.88	2.08	2.878(3)	151
Ow3—Hw32···O8	0.74(4)	2.08(4)	2.816(3)	173(4)
N32—H32A···O1	0.88	1.97	2.849(4)	172
N32—H32B···O7	0.88	2.03	2.896(4)	170
N33—H33A···Ow2	0.88	2.03	2.883(4)	162
N33—H33B···O8	0.88	2.03	2.904(3)	175
C2—H2B···Ow2	0.99	2.38	3.131(4)	132
C3—H3B···Ow3	0.99	2.49	3.476(4)	175

Table S3e. Hydrogen-bond geometry (Å, °) for $[C(NH_2)_3]_3[Er(EDTA)(F)_2]\cdot H_2O$

D—H···*A*	*D*—H	H···*A*	*D*···*A*	*D*—H···*A*
Ow—Hw1···O7	0.83(4)	1.91(4)	2.732(4)	174(3)
Ow—Hw2···F1	0.73(4)	1.99(4)	2.690(4)	163(4)
N21—H21A···O2	0.88	2.16	3.000(4)	160
N21—H21B···O8	0.88	2.04	2.895(4)	163
N22—H22A···F2	0.88	1.89	2.700(4)	153
N22—H22B···F1	0.88	1.88	2.729(3)	161
N23—H23A···O4	0.88	2.05	2.921(4)	170
N23—H23B···F2	0.88	2	2.822(4)	155
N31—H31A···F1	0.88	2.01	2.828(4)	155
N31—H31B···O6	0.88	2.16	2.918(4)	144
N32—H32A···N22	0.88	2.55	3.304(4)	145
N32—H32B···O5	0.88	2.08	2.888(4)	152
N33—H33A···O5	0.88	2.25	3.009(4)	145
N33—H33B···O6	0.88	2.01	2.808(4)	150
N41—H41A···O5	0.88	2.26	3.008(4)	143
N41—H41B···O3	0.88	2.15	2.916(4)	145
N42—H42A···Ow	0.88	1.94	2.785(4)	160
N42—H42B···O1	0.88	2	2.869(4)	168
N43—H43A···Ow	0.88	2.54	3.226(4)	136
N43—H43A···O7	0.88	2.62	3.460(4)	160
N43—H43B···O7	0.88	1.98	2.855(4)	175
C2—H2B···O8	0.99	2.52	3.133(4)	120
C3—H3B···N42	0.99	2.67	3.639(4)	165
C5—H5A···O5	0.99	2.68	3.618(4)	159
C7—H7B···Ow	0.99	2.69	3.384(4)	128
C9—H9A···O7	0.99	2.66	3.345(4)	127
C9—H9B···O6	0.99	2.39	3.379(5)	172

Table S3f. Hydrogen-bond geometry (Å, °) for $K_4[Er_2(EDTA)_2(F)_2]\cdot 4H_2O$

D—H···*A*	*D*—H	H···*A*	*D*···*A*	*D*—H···*A*
Ow—Hw1A···O5	0.78(5)	2.12(5)	2.866(4)	161(6)
Ow—Hw1B···O4	0.72(6)	2.16(5)	2.880(4)	173(6)
Ow2—Hw2A···Ow3	0.67(7)	2.14(7)	2.791(5)	169(7)
Ow2—Hw2B···Ow1	0.76(4)	2.67(4)	3.294(5)	140(4)
Ow2—Hw2B···O7	0.76(4)	2.44(4)	2.961(4)	127(4)
Ow3—Hw3A···Ow4	0.72(5)	2.03(5)	2.743(4)	177(9)
Ow3—Hw3B···O2	0.84(5)	2.63(5)	3.169(4)	123(4)
Ow3—Hw3B···O6	0.84(5)	1.92(5)	2.765(4)	178(6)
Ow4—Hw4A···Ow2	0.66(6)	2.10(5)	2.759(5)	179(7)
Ow4—Hw4B···O6	0.81(6)	1.98(6)	2.776(5)	169(5)
C1—H1B···Ow2	0.99	2.68	3.520(5)	143
C2—H2A···O6	0.99	2.59	3.314(4)	130
C3—H3A···Ow1	0.99	2.69	3.434(4)	133
C3—H3A···Ow3	0.99	2.6	3.205(4)	119
C3—H3B···Ow4	0.99	2.69	3.439(5)	132
C5—H5A···O7	0.99	2.35	3.325(4)	169
C9—H9A···O5	0.99	2.55	3.479(4)	156

Table S4. Oscillator strengths ($P\cdot10^8$) and Ω_λ parameter values for the crystals (colourless columns) and solutions (grey columns) (P values of hypersensitive transitions are in bold)

$^4I_{15/2} \rightarrow$	$[Er(EDTA)(H_2O)_2]^-$		$[Er(EDTA)F(H_2O)]^{2-}$		$[Er(EDTA)F_2]^{3-}$		$[Er(EDTA)(\mu\text{-}F)]_2^{4-}$		$[Er(EDTA)(\mu\text{-}OH)]_2^{4-}$		$[Er(EDTA)(H_2O)_x]^-$		$[Er(EDTA)(H_2O)_{x-1}(OH)]^{2-}$		$[Er(EDTA)(H_2O)_{x-1}F]^{2-}$	
	$P_{exp}\cdot10^8$	$P_{cal}\cdot10^8$	$P_{exp}\cdot10^8$	$P_{cal}\cdot10^8$	$P_{exp}\cdot10^8$	$P_{cal}\cdot10^8$	$P_{exp}\cdot10^8$	$P_{cal}\cdot10^8$	$P_{exp}\cdot10^8$	$P_{cal}\cdot10^8$	$P_{exp}\cdot10^8$	$P_{cal}\cdot10^8$	$P_{exp}\cdot10^8$	$P_{cal}\cdot10^8$	$P_{exp}\cdot10^8$	$P_{cal}\cdot10^8$
$^4I_{11/2}$	69.5	66.3	50.0	56.8	73.3	53.9	62.5	52.3	58.9	56.2	85.7	78.9	97.9	78.7		
$^4I_{9/2}$	17.5	32.8	13.5	30.7	22.7	31.3	19.1	37.5	16.8	34.9	19.3	37.0	24.7	41.7	18.2	37.5
$^4F_{9/2}$	222.6	224.7	164.1	199.7	167.7	198.5	199.6	213.1	188.2	213.5	239.0	255.8	241.3	267.7	213.1	241.4
$^4S_{3/2}$	56.3	62.3	63.0	52.2	57.7	49.0	52.8	45.2	34.9	50.8	77.0	71.3	80.5	68.2	70.8	61.8
**$^2H_{11/2}$**	298.1	323.4	302.9	341.3	311.4	343.3	472.0	485.7	397.0	386.2	477.5	520.3	625.7	670.7	438.0	480.3
$^4F_{7/2}$	234.4	239.0	208.2	203.8	194.9	195.2	186.5	192.3	215.4	205.5	272.1	272.9	268.2	268.7	247.3	243.3
$^4F_{5/2}$, $^4F_{3/2}$	117.6	119.1	106.0	99.5	95.4	93.6	87.6	86.2	107.3	96.8	131.9	136.3	127.5	129.9	124.7	117.8
$(^2G,^4F,^2H)_{9/2}$	90.7	91.2	78.5	77.0	79.3	72.7	77.2	69.1	96.2	76.0	99.3	104.4	99.0	100.9	93.0	91.4
**$^4G_{11/2}$**	584.1	570.2	621.6	599.6	624.9	606.8	869.2	861.4	677.6	682.8	943.9	920.1	1215.5	1190.2	869.0	844.6
$^4G_{9/2}$	154.5	144.9	179.6	131.4	173.3	130.7	172.8	147.2	175.8	143.0	192.6	164.7	216.5	176.4	199.5	158.6
$^2K_{15/2}$,$^2G_{7/2}$	112.2	99.2	79.0	86.5	66.9	82.4	76.9	84.8	50.2	87.0	129.8	120.3	116.2	123.1	89.8	105.9
$\Omega_2\cdot10^{20}$ [cm^2]		1.49±0.10		1.72±0.21		1.72±0.19		2.69±0.11		1.95±0.18		3.29±0.19		4.52±0.23		2.95±0.24
$\Omega_4\cdot10^{20}$ [cm^2]		1.55±0.16		1.46±0.33		1.50±0.29		1.82±0.18		1.67±0.28		1.98±0.30		2.25±0.36		2.02±0.39
$\Omega_6\cdot10^{20}$ [cm^2]		1.69±0.09		1.41±0.19		1.32±0.17		1.22±0.10		1.37±0.16		2.18±0.17		2.08±0.21		1.88±0.23
RMS$\cdot10^7$		1.32		2.48		2.77		1.48		2.35		2.23		2.70		2.84

Table S5. $(\sum \frac{\alpha}{r^4})^2$ factor for coordinated coligands in the Er(III)-EDTA complex and the corresponding Judd–Ofelt Ω_2 parameter values

	$(\sum \frac{\alpha}{r^4})^2$ / Å^{-2}	$\Omega_2 \cdot 10^{20}$ / cm^2
$K[Er(EDTA)(H_2O)_2]\cdot 4H_2O$	0.009196	1.49±0.10
$[C(NH_2)_3]_2[Er(EDTA)(H_2O)_2]ClO_4\cdot 6H_2O$	0.009085	2.11±0.24
$[C(NH_2)_3]_2[Er(EDTA)(F)(H_2O)]\cdot 2H_2O$	0.014525	1.72±0.21
$[C(NH_2)_3]_3[Er(EDTA)F_2]\cdot H_2O$	0.019716	1.72±0.19
$K_4[Er(EDTA)(\mu\text{-}F)]_2\cdot 8H_2O$	0.017258	2.69±0.11
$K_5[(Er(EDTA)(\mu\text{-}OH)]_2Cl\cdot 5.5H_2O$	0.028075	1.95±0.18
$[C(NH_2)_3]_3[Er(EDTA)(CO_3)]\cdot H_2O$	0.06849	4.96±0.14

α_{H_2O}=1.44 Å^3 [1], α_F=1.62 Å^3 [2], α_{OH}=2.26 Å^3 [2], $\alpha_{CO_3{}^{2-}}$=3.85 Å^3 [3]

[1] J. W. Rohleder, Polaryzowalność cząsteczek wody i anizotropia optyczna naturalnego gipsu, $CaSO_4\cdot 2H_2O$., *Ann. Soc. Chim. Pol.*, 1972, **46**, 2089–2097;

[2] R. D. Shannon, Dielectric polarizabilities of ions in oxides and fluorides., *J. Appl. Phys.* 1993, **73**, 348-366

[3] B. W. N. Lo, The polarizabilities of planar nitrate, carbonate and borate anions in crystal., *J. Phys. Chem. Solids*, 1973, **34**, 513–520.

Table S6a. Experimental data used for determination of osmotic coefficient and ε_γ parameter for Na^+ - $[Er(EDTA)]^-$ ionic pair

molality mol·kg^{-1}	osmolality	Φ_{exp}	Φ_{calc}
0.57497	0.9785	0.8509	0.8429
0.53917	0.9200	0.8532	0.8476
0.46722	0.8030	0.8593	0.8574
0.39086	0.6790	0.8686	0.8682
0.32566	0.5685	0.8728	0.8780
0.25886	0.4570	0.8827	0.8887
0.18855	0.3410	0.9043	0.9015
0.11777	0.2190	0.9298	0.9173
0.04765	0.0910	0.9550	0.9408
0.09104	0.1705	0.9364	0.9247
0.05926	0.1110	0.9365	0.9357
0.03537	0.0680	0.9613	0.9471
0.02743	0.0523	0.9538	0.9521
0.01255	0.0240	0.9562	0.9651
0.00629	0.0122	0.9701	0.9740
0.58008	0.9800	0.8447	0.8422
0.50366	0.8620	0.8557	0.8524
0.43949	0.7570	0.8612	0.8613
0.36050	0.6250	0.8668	0.8727
0.28430	0.4995	0.8785	0.8845
0.22110	0.3960	0.8955	0.8954
0.15068	0.2750	0.9125	0.9094
0.08000	0.1505	0.9407	0.9282

Table S6b. Experimental data used for determination of osmotic coefficient and ε_γ parameter for K^+ - $[Er(EDTA)]^-$ ionic pair

molality mol·kg^{-1}	osmolality	Φ_{exp}	Φ_{calc}
0.06240	0.1160	0.9295	0.9330
0.10210	0.1880	0.9207	0.9190
0.14173	0.2577	0.9090	0.9080
0.18141	0.3260	0.8985	0.8986
0.21958	0.3890	0.8858	0.8903
0.26217	0.4630	0.8830	0.8819
0.30228	0.5267	0.8711	0.8743
0.34209	0.5890	0.8609	0.8672
0.38160	0.6550	0.8582	0.8603
0.42074	0.7203	0.8560	0.8538
0.22255	0.3990	0.8964	0.8897
0.18370	0.3310	0.9009	0.8981
0.15647	0.2850	0.9107	0.9044
0.12866	0.2350	0.9133	0.9114
0.10704	0.1970	0.9202	0.9175
0.08261	0.1520	0.9200	0.9254

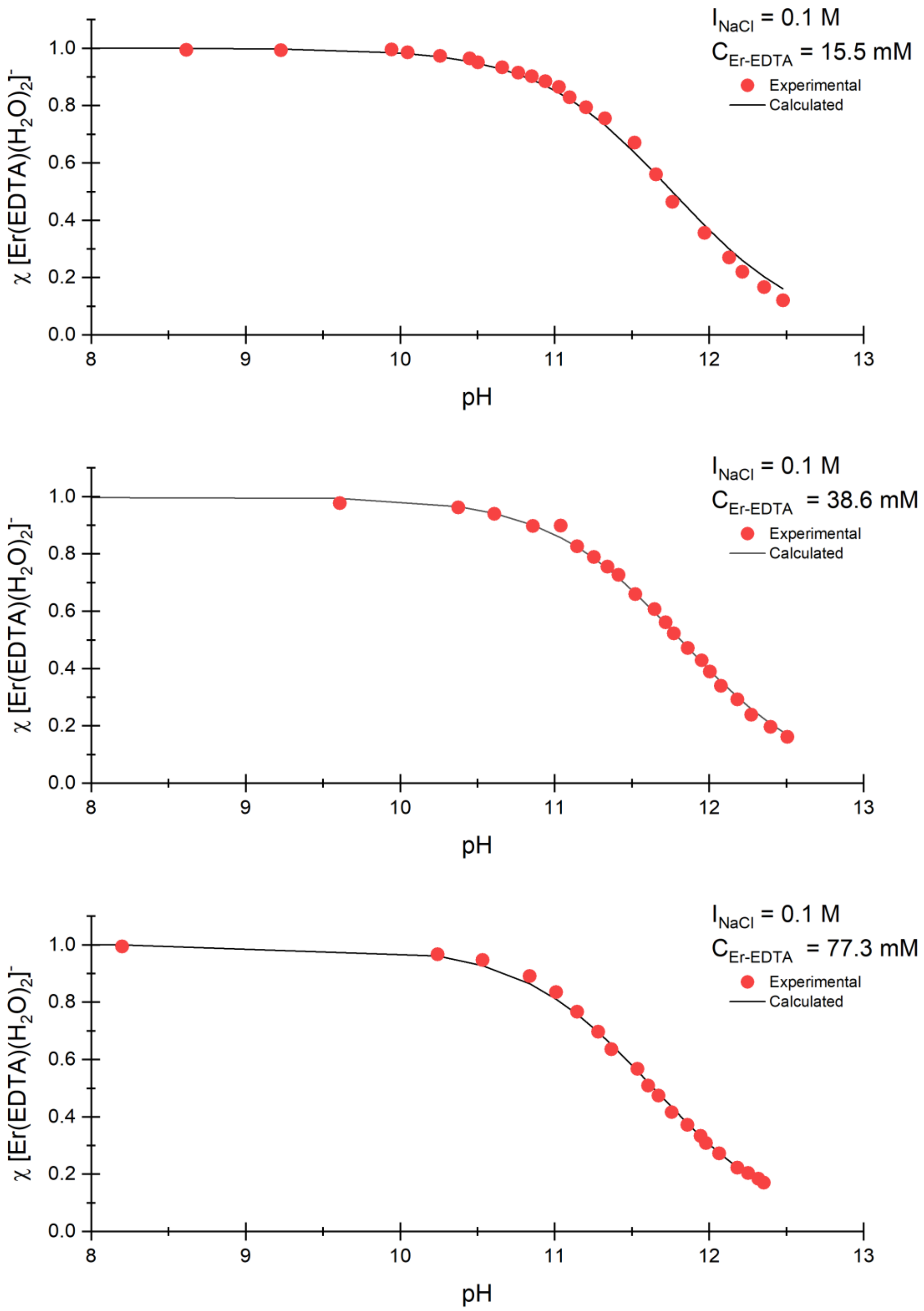

Figure S1. Experimental (•) and calculated (—) mole fractions of $[Er(EDTA)(H_2O)_2]^-$ species as a function of pH in NaCl electrolyte.

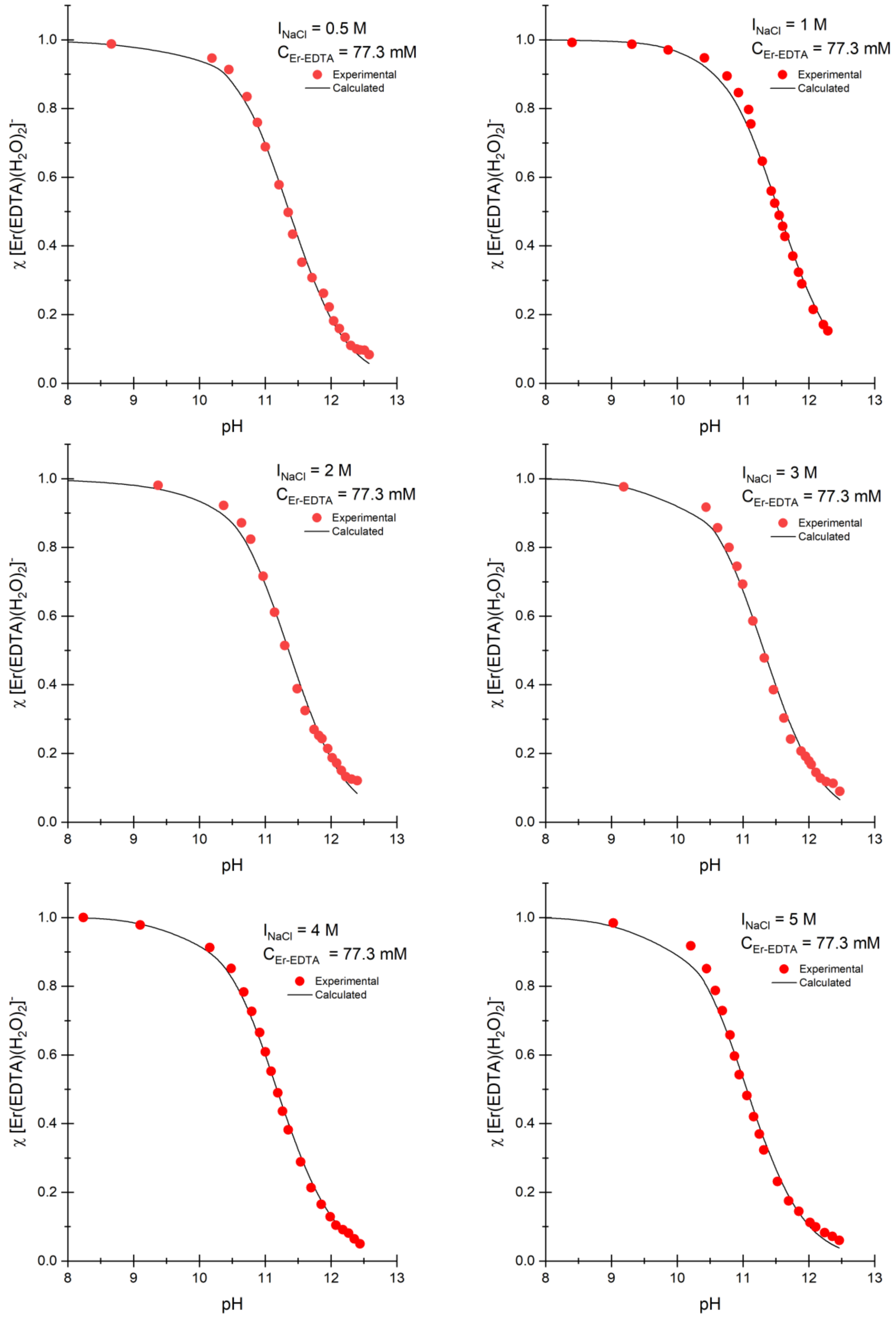

Figure S2. Experimental (•) and calculated (—) mole fractions of $[Er(EDTA)(H_2O)_2]^-$ species as a function of pH in NaCl electrolyte.

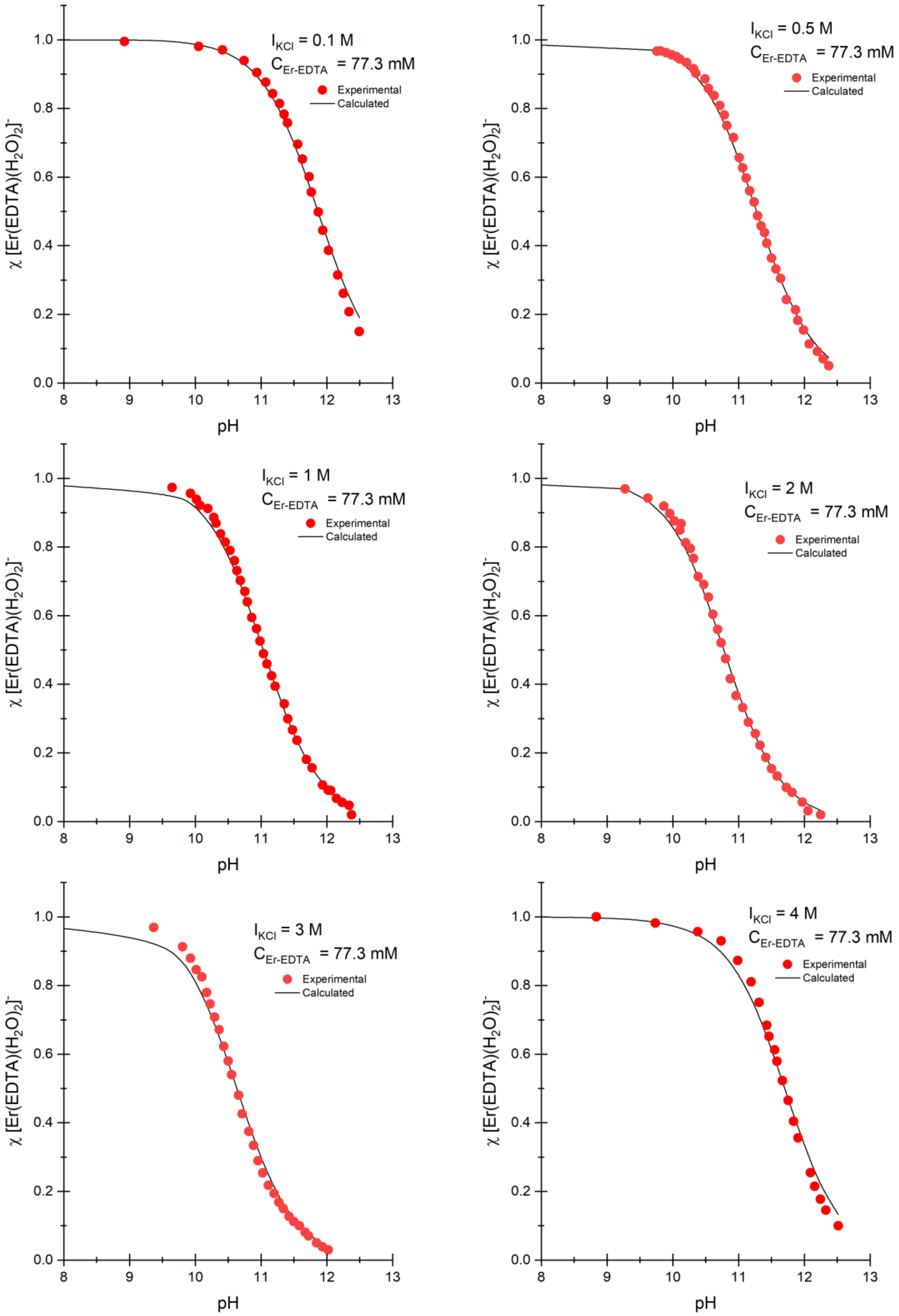

Figure S3. Experimental (•) and calculated (—) mole fractions of $[Er(EDTA)(H_2O)_2]^-$ species as a function of pH in KCl electrolyte.

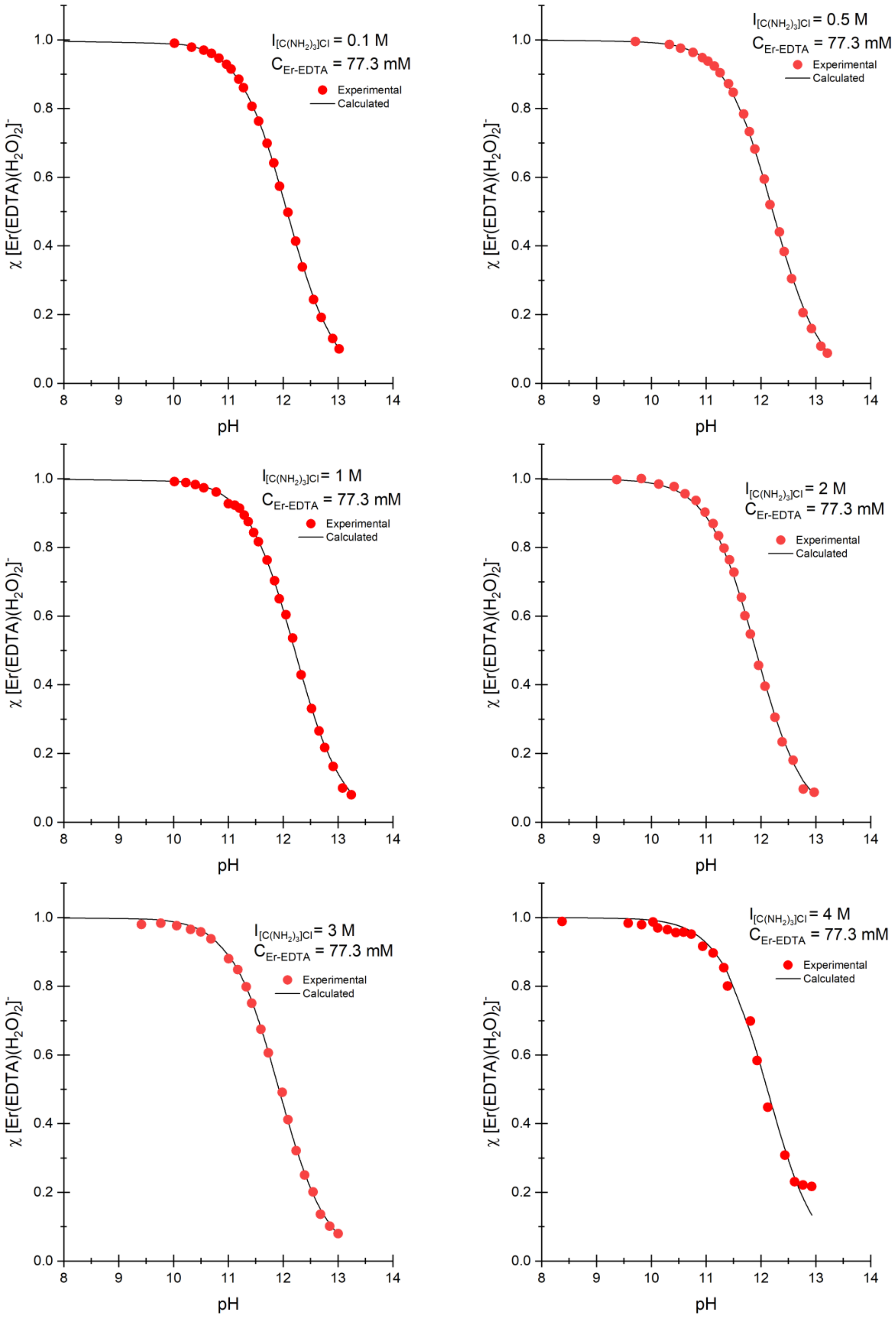

Figure S4. Experimental (•) and calculated (—) mole fractions of $[Er(EDTA)(H_2O)_2]^-$ species as a function of pH in $[C(NH_2)_3]Cl$ electrolyte.

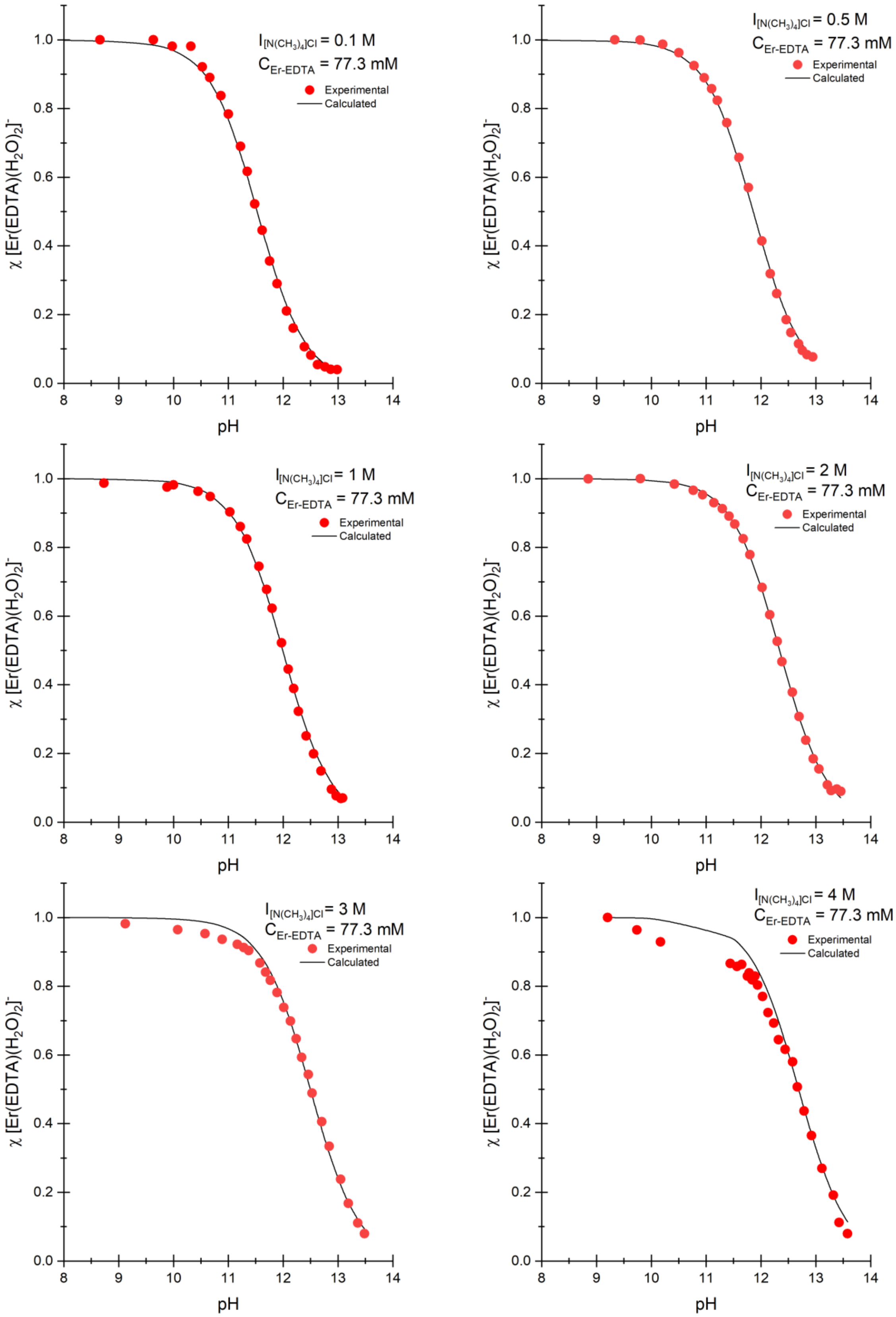

Figure S5. Experimental (•) and calculated (—) mole fractions of $[Er(EDTA)(H_2O)_2]^-$ species as a function of pH in $[N(CH_3)_4]Cl$ electrolyte.

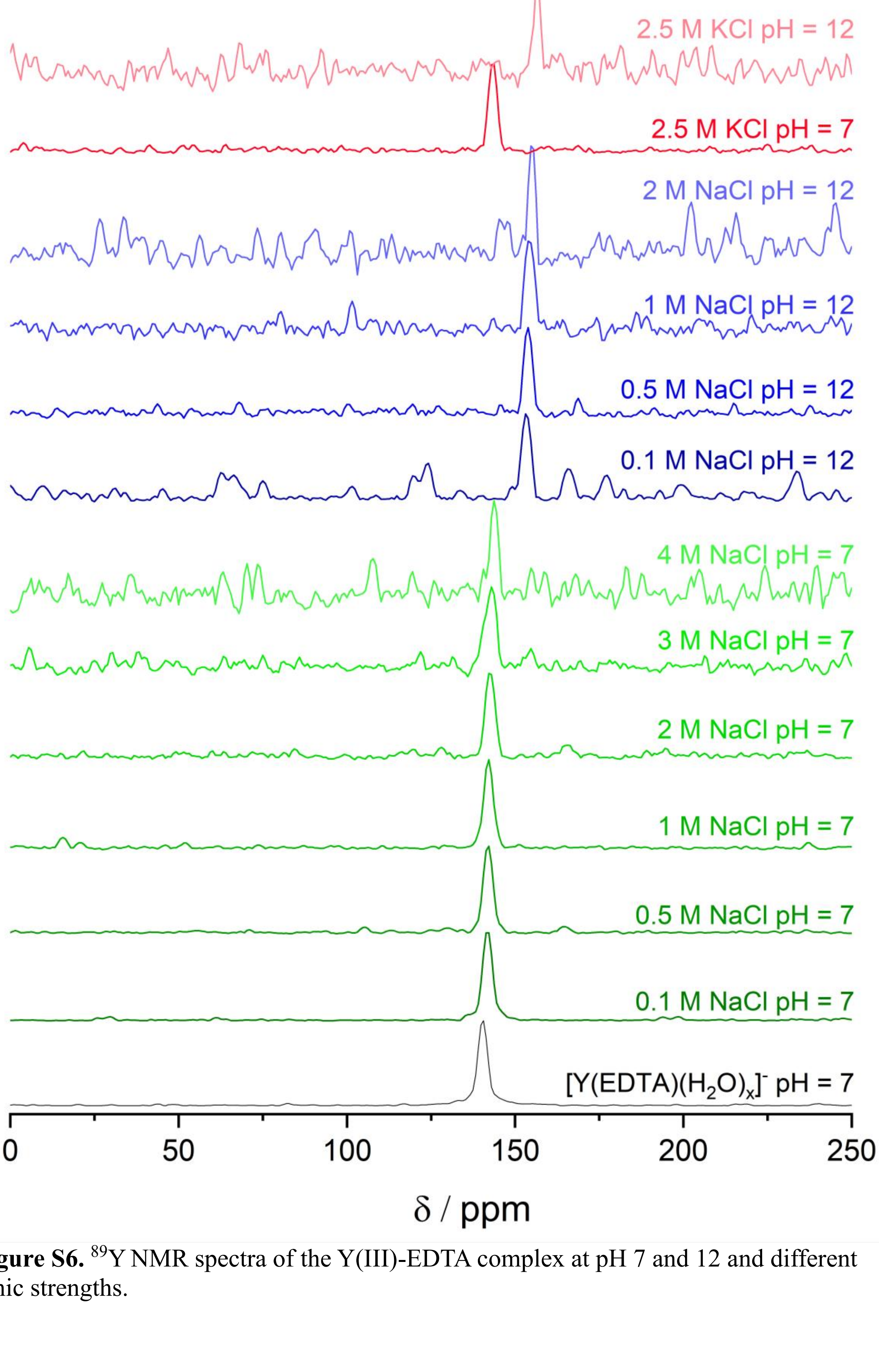

Figure S6. ^{89}Y NMR spectra of the Y(III)-EDTA complex at pH 7 and 12 and different ionic strengths.

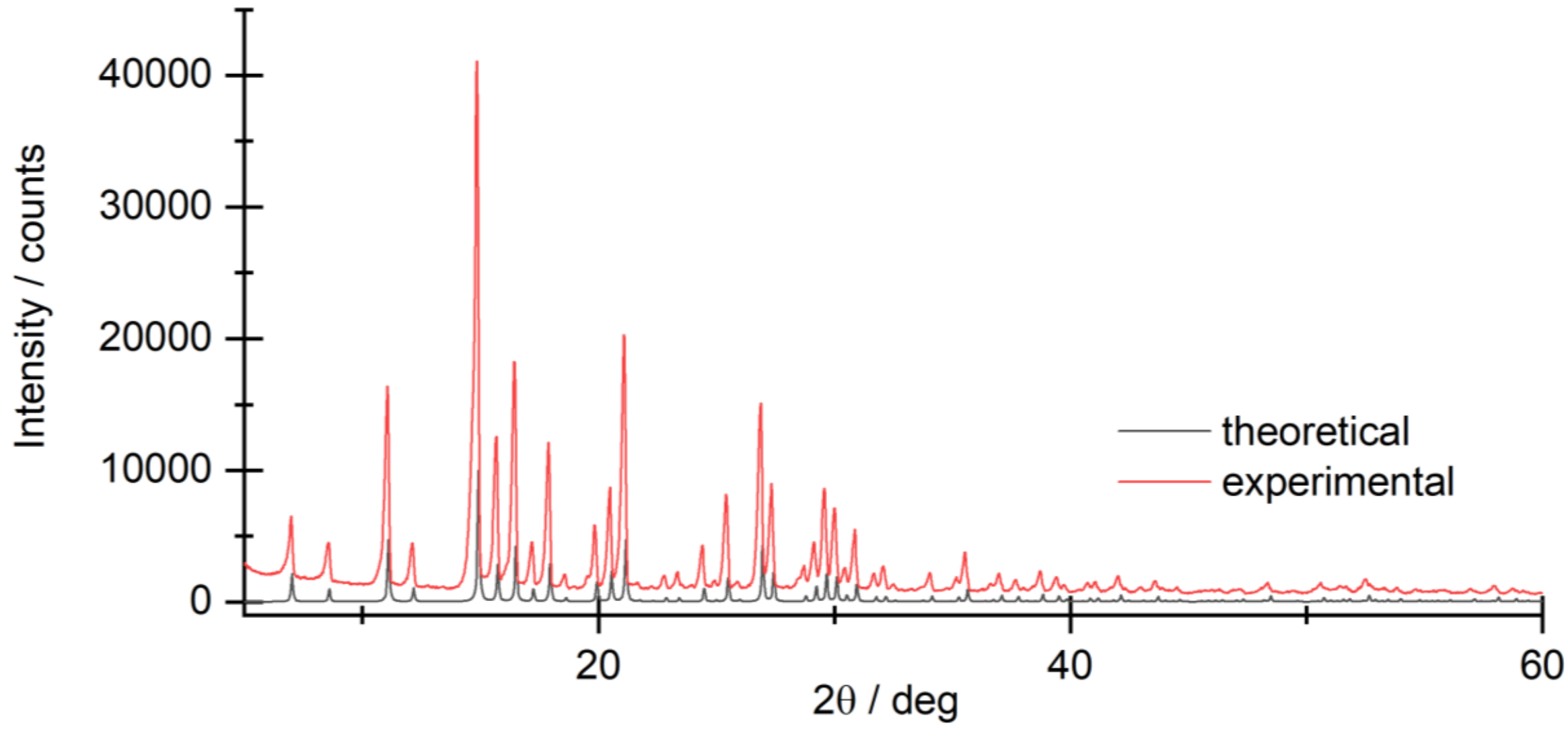

Figure. S7. Theoretical and experimental X-ray powder diffractogram of the $[C(NH_2)_3]_2SO_4$ crystal. Experimental recorded at RT, theoretical generated for solved structure measured at 100 K.

Figure. S8. EDS spectra of the Er2OH2.

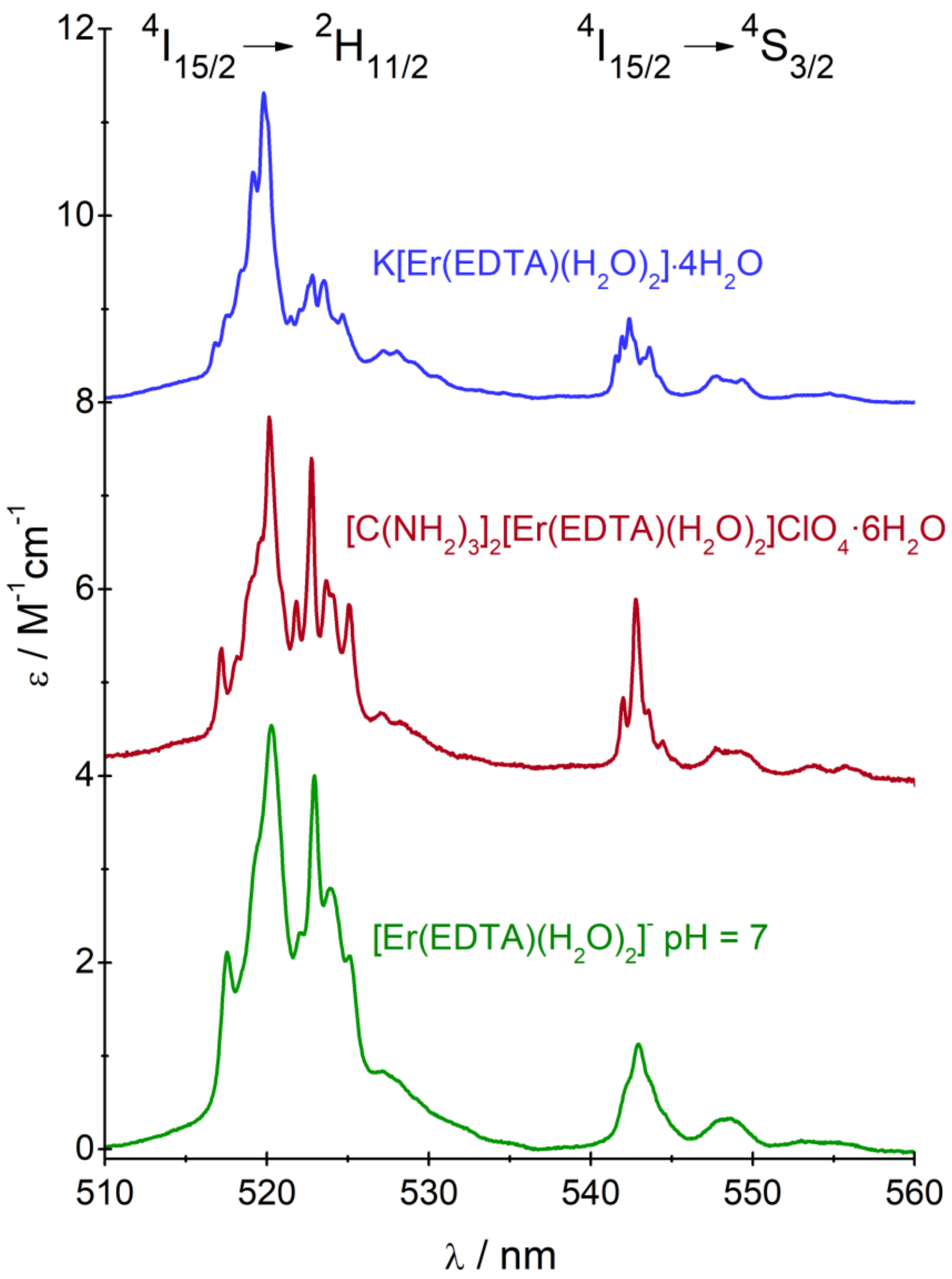

Figure S9. Absorption spectra of the 4$I_{15/2} \rightarrow {}^2H_{11/2}$ and 4$I_{15/2} \rightarrow {}^4S_{3/2}$ recorded for K[Er(EDTA)(H_2O)$_2$]·4H_2O and [C(NH_2)$_3$]$_2$[Er(EDTA)(H_2O)$_2$]ClO_4·6H_2O crystals and [Er(EDTA)(H_2O)$_2$]$^-$ solution at pH = 7.

The comparison of the solid-state and solution-state UV-vis spectra is based on the overall similarity of the f-f transition patterns, particularly the positions and general distribution of the absorption bands in the relevant spectral regions. While an exact one-to-one correspondence of individual transitions is not expected for Er(III) due to the complexity of its electronic structure, the observed spectral similarities suggest that the coordination environment of the Er(III) center in solution is consistent with that in the solid state.

To illustrate the complexity of relating solid-state and solution spectra, the electronic absorption spectra ($^4I_{15/2} \rightarrow {}^2H_{11/2}$ $^4I_{15/2} \rightarrow {}^4S_{3/2}$ transitions) of the [Er(EDTA)(H_2O)$_2$]$^-$ complex recorded in aqueous solution (pH = 7) and in two single-crystals, [C(NH_2)$_3$]$_2$[Er(EDTA)(H_2O)$_2$]ClO_4·6H_2O (previously published by us) [4] and K[Er(EDTA)(H_2O)$_2$]·4H_2O, were compared. Importantly, both crystals contain the same

coordination $[Er(EDTA)(H_2O)_2]^-$ species, differing primarily in the symmetry of the complex: C_1 in $[C(NH_2)_3]_2[Er(EDTA)(H_2O)_2]ClO_4 \cdot 6H_2O$ crystal and exact C_2 in $K[Er(EDTA)(H_2O)_2] \cdot 4H_2O$ crystal. Nevertheless, some differences in the spectral fine structure are observed between the two crystalline forms. These differences include spectral pattern and intensity of f-f bands. At the same time, both spectra retain the characteristic features expected for the same coordination environment of the Er(III). Interestingly, the solution spectrum exhibits a closer resemblance to that of the lower-symmetry complex - C_1 in $[C(NH_2)_3]_2[Er(EDTA)(H_2O)_2]ClO_2 \cdot 6H_2O$ crystal. However, the spectrum of the higher-symmetry complex - C_2 in $K[Er(EDTA)(H_2O)_2] \cdot 4H_2O$ crystal, cannot be regarded as fundamentally different despite its lower degree of similarity to the solution spectrum. These observations indicate that substantial spectral variations may arise from differences in symmetry and crystal-field effects even when the composition and coordination mode of the complex remain unchanged. Consequently, exact spectral coincidence should not be regarded as a prerequisite for concluding that similar coordination environments are present in the solid state and in solution. In other words, the lack of perfect correspondence between the spectra does not exclude the possibility that Er(III) complexes possess the same coordination environment in the crystal and in solution. Moreover, we have shown that differences between the spectra of solid-state samples and solutions are often caused by dynamic effects. This was studied and discussed by Bukietyńska et al. [5] in the context of Judd–Ofelt parameter values. Only in the case of very rigid structures, such as the $[Eu(DOTP)]^{5-}$ complex [6], is there an almost 1:1 correspondence between the spectra in the solid state and in solution.

[4] Janicki R., Mondry A., A new approach to determination of hydration equilibria constants for the case of $[Er(EDTA)(H_2O)_n]^-$ complexes., *Phys. Chem. Chem. Phys.,* 2014, **16**, 26823-26831.

[5] K. Bukietyńska, A. Mondry, P.N. Thuy, P. Starynowicz, The role of molecular dynamics in the ff transition intensities in lanthanide compounds. *Journal of Alloys and Compounds*, 1995, **225(1-2)**, 52-54

[6] R. Janicki, A. Kędziorski, A. Mondry Experimental and Ab Initio Study on the Intensities of f–f Transitions for the Molecular Eu(III)-DOTP System, *Chemistry Select*, 2019, **4**, 1394 – 1402

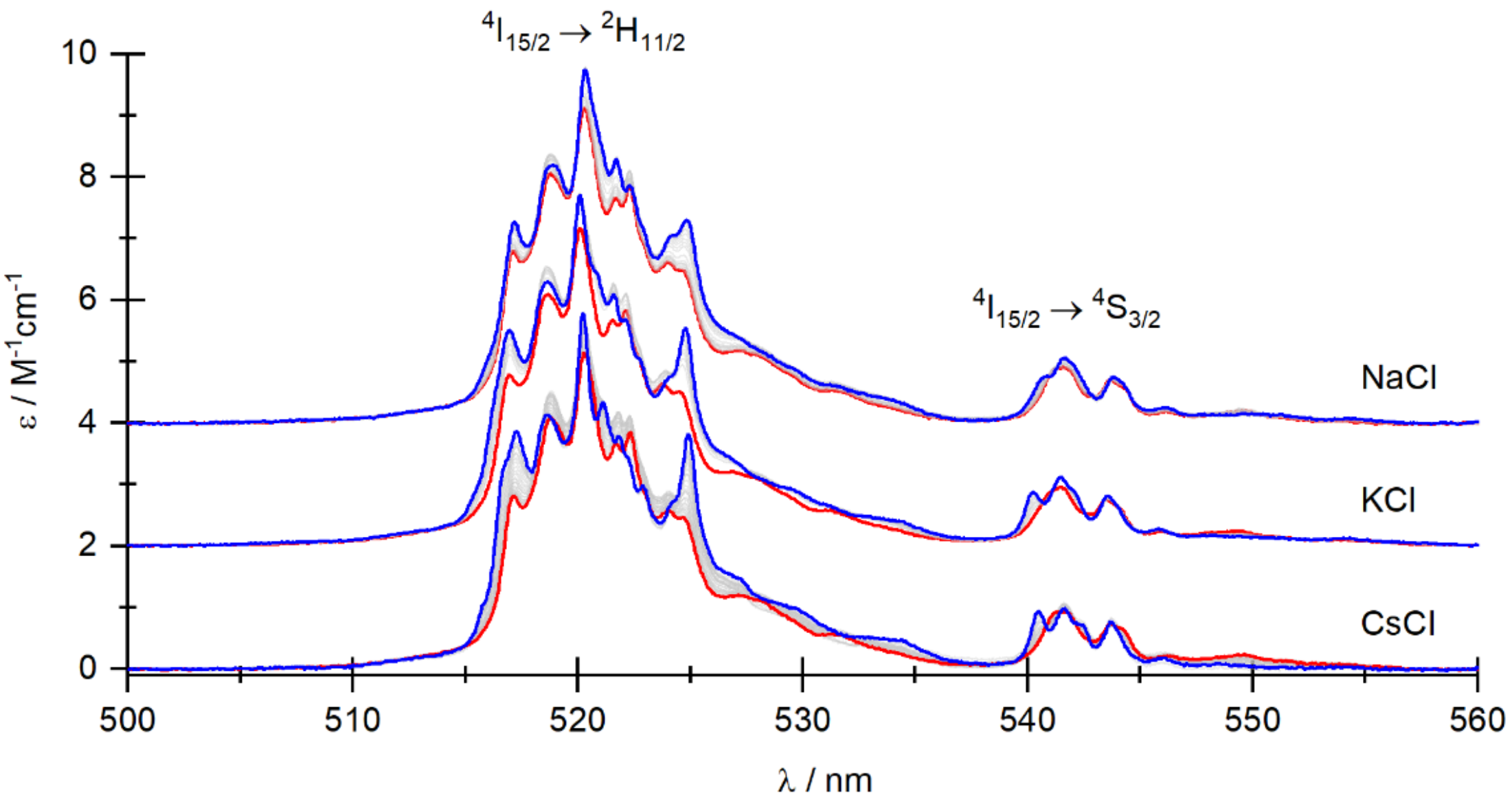

Figure S10. Absorption spectra (initial – red, final – blue) of the $^4I_{15/2}\rightarrow{}^2H_{11/2}$ and $^4I_{15/2}\rightarrow{}^4S_{3/2}$ transitions of the Er(III)-EDTA system recorded during gravimetric titrations of the basic solutions of Er(III)-EDTA-OH with solid NaCl, KCl and CsCl.

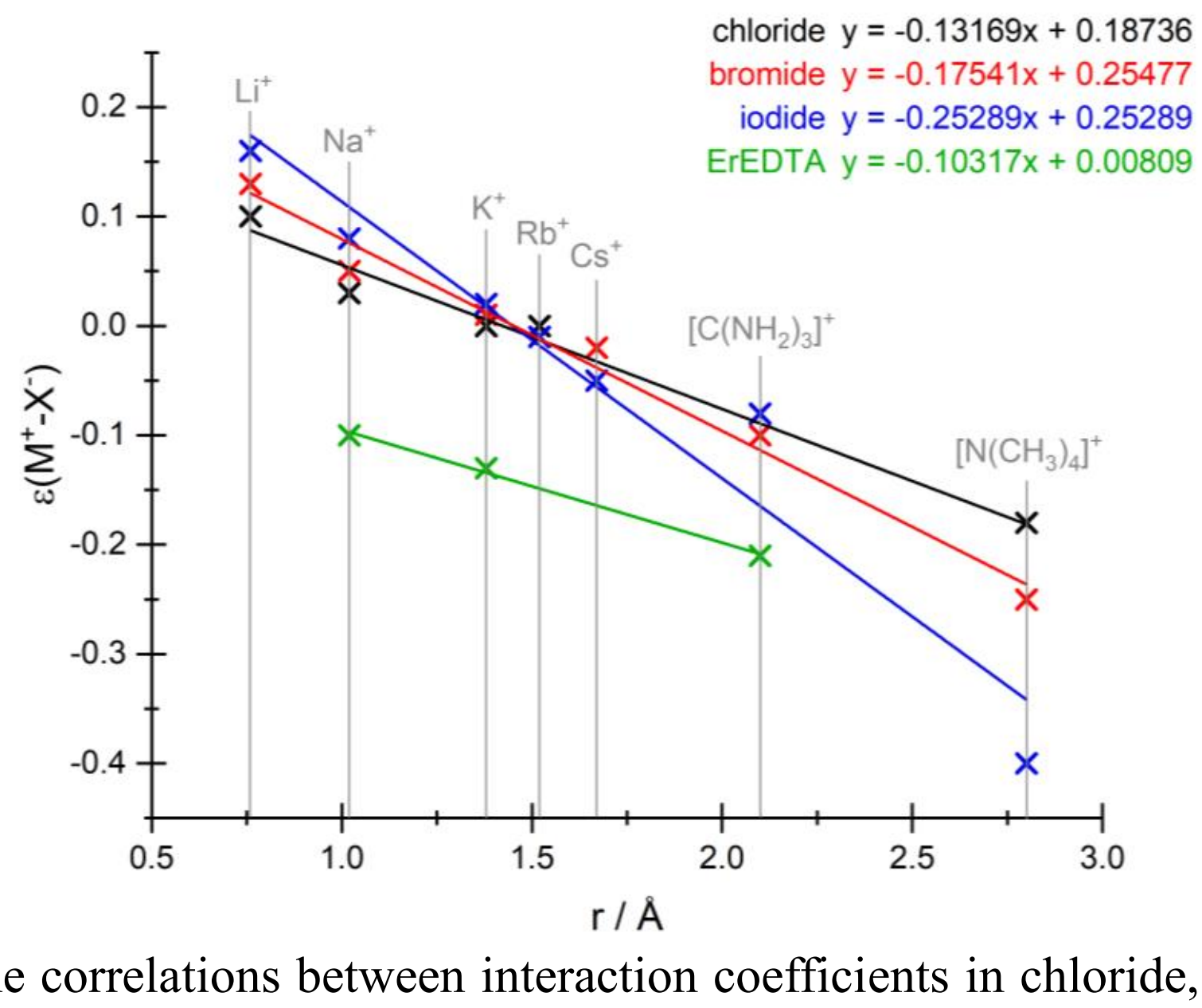

Figure. S11. The correlations between interaction coefficients in chloride, bromide, iodide and $[Er(EDTA)]^-$ media for monovalent cations.

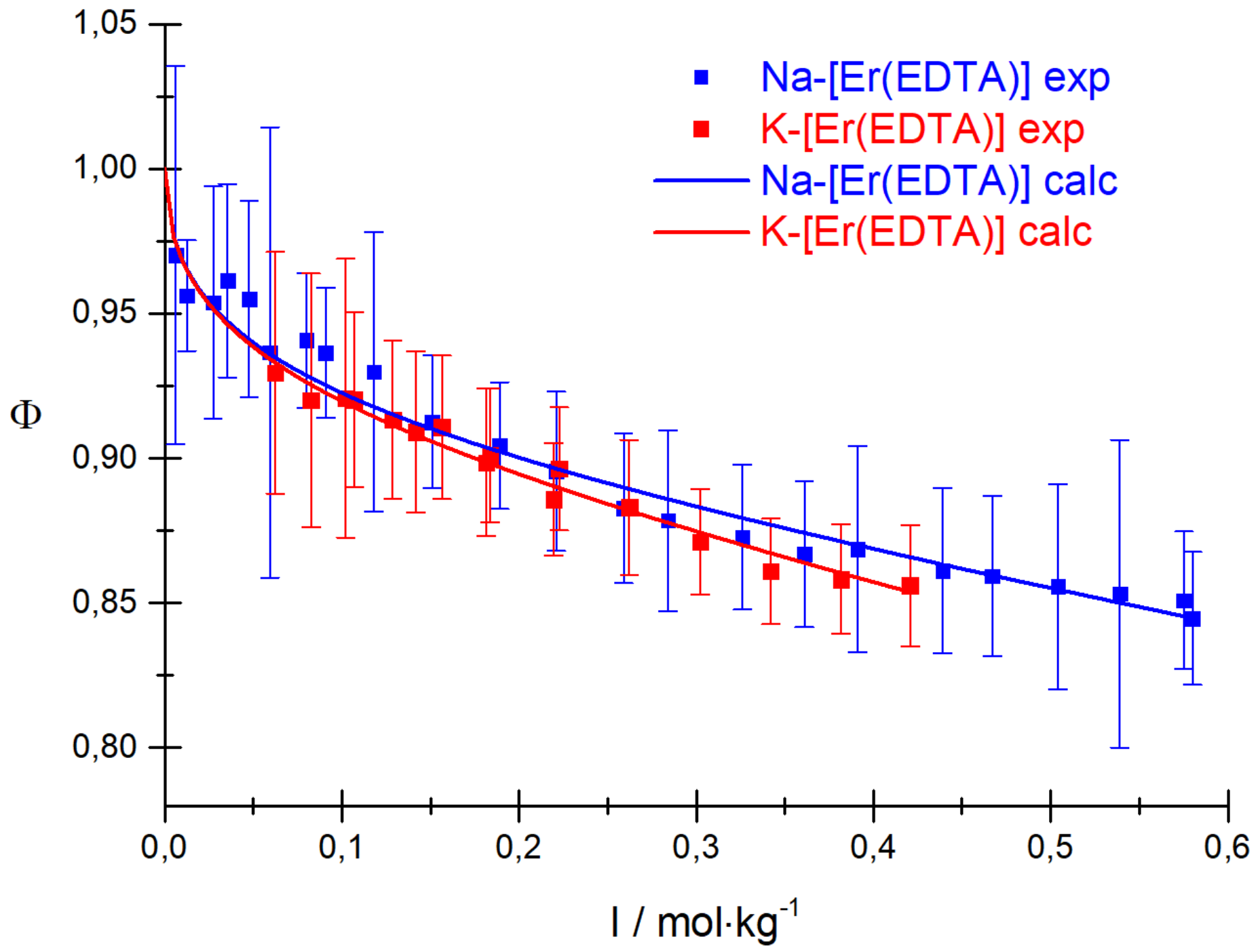

Figure S12. Plot of osmotic coefficient vs. ionic strengths for Na-Er(EDTA) and K-Er(EDTA) solutions.
